# Enhancing the Generalizability of Deep Learning–Based Models for Lung Field Segmentation in Chest Radiographs Using Edge‐Assisted Multiscale Feature Fusion

**DOI:** 10.1155/ijbi/6566262

**Published:** 2026-05-07

**Authors:** Tairah Andrabi, Sajid Yousuf Bhat

**Affiliations:** ^1^ Department of Computer Science, University of Kashmir, Srinagar, Jammu and Kashmir, India, kashmiruniversity.net

**Keywords:** CXR image, deep learning, edge features, feature fusion, lung field segmentation, morphology

## Abstract

**Introduction:**

Lung field segmentation (LFS) in chest x‐rays (CXR) is a key step for the computer‐assisted diagnosis of respiratory diseases. However, achieving precise delineation remains challenging in images with poor contrast and superimposed anatomical structures, often resulting in imprecise lung outlines in deep learning (DL) models. Furthermore, existing models often lack generalizability, performing well on specific datasets but failing on unseen domains due to variations in imaging equipment, acquisition protocols, and patient demographics.

**Methods:**

To overcome these limitations, we propose a two‐phase hybrid approach for robust and generalizable LFS in CXR. The first phase employs a heuristic approach to extract and refine multiscale edge features: The Laplacian of Gaussian (LoG) filter detects closed contours that effectively highlight the overall lung shape, whereas the Canny detector captures the finer, high‐frequency edge details that preserve the lung outlines often missed by DL models due to overlapping structures. In the second phase, the enriched multiscale feature maps are fused along with the original contrast inputs and then used to train and evaluate various U‐Net architectures.

**Results:**

The proposed approach is evaluated on three benchmark datasets: Montgomery County (MC), Shenzhen Hospital (SH), and Japanese Society of Radiological Technology (JSRT). Experiments conducted on individual datasets using five‐fold cross‐validation and testing on a completely unseen separate test set to simulate real‐world settings revealed that the proposed edge‐assisted filters, especially the Canny filter, significantly improve segmentation performance metrics. Among all U‐Net variants, deep attention U‐Net achieved the highest performance using a consistent channel combination, attaining a dice coefficient of 0.9815, a Jaccard score of 0.9624 on the JSRT dataset. The proposed approach achieved a dice gain of +0.0011 to +0.0034 and an IoU gain of +0.009 to +0.0034 across the three datasets compared with the baseline configuration using original images. Furthermore, cross‐dataset validation also depicted improvement gains in dice, IoU, and other metrics, demonstrating the generalizability of the proposed approach.

**Conclusion:**

Further cross‐dataset validation confirmed the framework′s strong generalization capabilities, demonstrating stable performance even on unseen domains. These findings validate the proposed method as a reliable and effective solution for LFS in diverse and clinically challenging environments, offering promising benefits for respiratory diagnosis and patient care.

## 1. Introduction

Lung diseases are one of the major global concerns, affecting the respiratory system through a broad spectrum of conditions ranging from infectious diseases such as asthma, tuberculosis, and pneumonia, to chronic conditions like emphysema and COPD, as well as life‐threatening diseases like lung cancer and COVID‐19. Early and accurate detection of these illnesses plays a vital role in effective treatment. Healthcare professionals rely on various medical imaging modalities such as “chest x‐rays (CXR), magnetic resonance imaging (MRI), and computed tomography (CT) scans” [1]. These scans provide a visual representation of the lungs, heart, ribs, and other adjacent tissues. Among these, x‐ray imaging remains the most commonly used modality due to its low cost, widespread availability, and minimal radiation exposure [2]. Despite its widespread use, manual interpretation of x‐rays is challenging and requires expertise to accurately identify abnormalities and differentiate them from normal anatomical structures [3]. This highlights the development of computer‐aided diagnosis (CAD) systems, which provide valuable assistance to radiologists by automating image analysis and improving diagnostic accuracy and timely decision‐making.

A fundamental preprocessing step in CAD systems is medical image segmentation, which is aimed at isolating the region of interest (RoI), enabling focused analysis of relevant areas. This computer vision task of segmentation can be either “single‐organ segmentation” or “multiorgan segmentation”. In single‐organ segmentation, the focus is on identifying and isolating a specific organ or tissue from medical images. This plays a crucial role in clinical tasks such as diagnosing lung tumors, detecting skin lesions, or identifying brain tumors. On the other hand, multiorgan segmentation involves segmenting several organs within the same image simultaneously, such as in the abdominal region, where the pancreas, liver, and gallbladder interact closely, or in the pelvic region, where organs like the uterus, prostate, and bladder are closely related. In the context of chest radiography, lung segmentation is particularly important, as accurate delineation of lung regions forms the foundation for tasks like pulmonary disease detection and severity assessment. However, precise segmentation of lung fields itself poses considerable challenges in chest x‐rays due to several inherent factors, such as (i) anatomical variations in lung shape and size affected by factors like gender and age, and (ii) the presence of overlapping organs like the ribcage and clavicles, which can obscure lung boundaries [4]. These challenges hinder the accurate delineation of lung boundaries and thus impact diagnosis. This highlights the need for an innovative segmentation approach that can effectively address the complexities of lung field segmentation (LFS) and provide reliable results across diverse chest x‐ray datasets.

Traditional lung segmentation approaches primarily relied on rule‐based techniques, pixel‐classification (PC)–based, deformable‐based models [5]. These methods explicitly exploit anatomical and structural priors and are generally training‐free, making them computationally efficient and independent of annotated data. However, such methods are often sensitive to noise and parameter tuning, limiting robustness across diverse datasets. In contrast, recent advances in deep learning (DL), particularly convolutional neural networks (CNNs) and U‐Net–based architectures, have significantly improved segmentation accuracy as they automatically extract the features, boosting the diagnostic precision and treatment efficiency. Although these models have shown strong performance on benchmark datasets, their generalizability to unseen data remains a critical concern.

Most DL based lung segmentation models are trained and evaluated on the same dataset using a fixed train‐test split. This evaluation strategy often produces high‐performance metrics that do not adequately reflect the model′s ability to generalize to unseen data. Consequently, their performance frequently degrades when applied to CXR images acquired under different hardware setups, imaging protocols, and patient demographics, which can compromise the model′s consistency. This limitation highlights the need for evaluation strategies that emphasize cross‐dataset validation and generalizability rather than single‐dataset optimization.

Motivated by the limitations of existing DL‐based LFS methods, particularly their generalizability across datasets, this study proposes a novel two‐phase approach that combines heuristic image processing with a DL‐based segmentation approach. The first phase does not rely on training and testing datasets, thus eliminating the need for extensive data annotation and model training. In the first phase, edge detection techniques, including the Canny edge detector for capturing the fine boundary details and the Laplacian of Gaussian (LoG) operator for detecting edges at multiple scales, are employed. These edge representations are further refined through morphological processing and pruning to ensure boundary continuity while suppressing the irrelevant surrounding anatomical structures. These edge‐assisted representations form the basis for the second phase of the study, which focuses on systematic evaluation of DL‐based LFS models. In this phase, multiple U‐Net–based segmentation models are analyzed by training and testing them using both original CXR images and edge‐assisted images. This comparative analysis enables a clear assessment of the impact of edge‐assisted features on segmentation metrics and cross‐domain generalizability.

The novelty of the proposed study lies in the integration of anatomically guided edge‐assisted features trained using DL based segmentation models to enhance the lung boundary localization without relying on additional postprocessing steps. The two‐phase strategy is particularly effective in improving segmentation performance for challenging lung boundaries that are difficult to delineate using purely data‐driven methods. The primary contributions of this study are outlined below:

1. To conduct a comprehensive review of existing LFS approaches using CXR images with particular emphasis on DL, highlighting their strengths, limitations, and clinical relevance.
2. To develop a training‐free, annotation‐independent heuristic edge extraction and refinement approach to extract features representing lung fields to enhance the region the localization prior to segmentation.
3. To conduct a detailed comparative evaluation of U‐Net and its multiple variants, integrated with edge‐assisted features, trained using K‐fold cross‐validation within benchmark CXR datasets, in order to analyze their impact on segmentation performance.
4. To evaluate the generalizability of the proposed approach through cross‐dataset validation and systematic ablation experiments, aimed at quantifying the individual contribution of the heuristic in terms of dice similarity coefficient (DSC) and Jaccard score (IoU), and performing comparative analysis against state‐of‐the‐art (SOTA) segmentation methods.

The present study is guided by the following research questions (RQs):

RQ1: Can a multiscale heuristic edge refinement provide anatomically meaningful priors that enhance the performance of DL‐based LFS models?

RQ2: Why does the standard U‐Net highly benefit most from these edge‐assisted feature fusions, as compared with other architectures within the dataset, as well as in cross‐dataset validation?

RQ3: Does the incorporation of multichannel edge‐assisted representations improve performance in terms of DSC and Jaccard score compared with baseline original images under cross‐dataset validation?

The rest of the article is structured as follows: Section II, “Related Work”, covers the existing literature related to lung segmentations. Section III, “Proposed Method,” explains the novel approach and methodology. Section IV describes the experimental setup covering the datasets, training strategy, and evaluation metrics used in this study. Section V presents the segmentation results obtained by evaluating DL models using public CXR datasets. Section VI compares the proposed method and existing methods in the literature. Section VII, “Discussion,” goes through the main findings and insights. Section VIII addresses the limitations of this research and possible directions of future research. Lastly, Section IX, “Conclusion,” summarizes the paper by concluding about the key contributions.

## 2. Related Work

Research on lung segmentation using CXR images has expanded rapidly in recent years, with the growing adoption of DL‐based techniques in medical imaging. Numerous studies in this domain primarily are aimed at delineating anatomical structures such as lungs, clavicles, and disease‐related patterns from CXR images. To review existing contributions, this section categorizes prior work into two major groups. The first group focuses exclusively on segmentation‐based approaches for precise localization. The second group includes segmentation‐guided classification approaches, where segmentation results are utilized to enhance the disease classification performance.

### 2.1. Segmentation‐Based Approaches

Deep CNNs and their variants have emerged as the dominant approach for LFS in CXRs. In particular, encoder–decoder architectures like the U‐Net model, originally proposed by Ronneberger et al. [6], have demonstrated remarkable effectiveness in medical image segmentation tasks. Accurate and automatic delineation of RoI in CXR images is essential for supporting the diagnosis of various pulmonary diseases. Several studies have proposed enhanced U‐Net–based models to improve the segmentation performance. Liu et al. [7] proposed a modified U‐Net–based architecture that utilizes a pretrained EfficientNet‐B4 backbone as the encoder. To enhance the feature learning, residual connections along with the leaky ReLU activation functions were incorporated in the decoder. The proposed model was evaluated on the two public datasets, namely the Montgomery County (MC) and Japanese Society of Radiological Technology (JSRT), as well as a private Haut dataset. Experimental results achieved notable performance improvements compared with the standard U‐Net. With the modifications in the standard U‐Net, Nishio et al. [8] utilized Bayesian optimization to fine‐tune a U‐Net for lung segmentation in CXRs with severe abnormalities, achieving improved dice scores on resized and equalized images from JSRT, MC, and NIH datasets. Likewise, Ray et al. [9] introduced XR‐U‐Net, an enhanced DL model derived from the U‐Net for lung segmentation in CXR images. The key modification lies in the adoption of a five‐stage encoder–decoder model that facilitates deeper feature extraction without the need for additional preprocessing. The effectiveness of the method was evaluated on the MC dataset, where it achieved an accuracy of 95.7%, along with a DSC of 91% and a Jaccard score of 83%. However, the study is limited to a single dataset evaluation, and further validation on more diverse datasets was suggested to strengthen the model′s generalizability. Training DL models from scratch often results in slow convergence and overfitting. Pyar [10] proposed a transfer learning–based pneumonia segmentation approach using DeepLabV3 and SegNet architecture trained and evaluated on the Kermany CXR dataset to precisely identify the pneumonia region localization. The performance of the model was assessed using segmentation‐specific metrics: Accuracy and IoU, and the results revealed that DeepLabV3 outperformed SegNet, by achieving an accuracy of 0.844 and an IoU of 0.81. Based on these findings, the authors suggested future work focusing on the integration of ensemble models to further improve segmentation performance. Generative adversarial networks (GANs) have also contributed significantly; Munawar et al. [11] used a U‐Net–based GAN for refined lung mask generation, whereas others [12, 13] extended GANs to multiorgan segmentation with high accuracy. More recently, transformers [14] and multiattention mechanisms have further improved segmentation by modelling long‐range dependencies and global context, boosting generalizability across diverse CXR datasets. To analyze the impact of attention mechanisms, Wahyuningrum et al. [15] proposed a “fully convolutional attention network (FCA‐NET)” architecture for segmenting the lungs from CXR images. Their approach incorporated both spatial and channel attention modules into a ResNetv2‐based encoder to precisely delineate the lung boundaries and enhance the multiscale feature extraction. The model was trained on a public CXR dataset from Qatar University, comprising 1500 CXR images of patients with COVID‐19, normal, and pneumonia cases. The study adopted K‐fold CV, varying K from 2 to 10; their optimal configuration achieved a Jaccard score of 94.66% and a dice score of 97.24% at *k* = 5, outperforming the existing SOTA methods such as SegNet, DeepLabV3, and UNet++. Further extending attention‐based segmentation approaches, Alam et al. [16] introduced AMRU++, which incorporated multiscale residual blocks and attention gates to enhance the contextual feature representation in CXR images. To improve the segmentation performance, the authors proposed a data augmentation technique referred to as selective cut‐out, and the model achieved superior dice and Jaccard scores across multiple public and private datasets. Moving beyond CNN‐based approaches, recent studies, Khorasani and Mofrad [17], introduced FAT‐Net, a transformer‐based architecture designed to capture both local and spatial features in CXR images. The model was validated on combined Shenzhen Hospital (SH) and MC datasets, comprising a total of 704 CXR radiographs, and achieved 98.12% accuracy and a dice of 96.10%, surpassing the performance of attention U‐Net and SegNet models.

### 2.2. Segmentation‐Guided Classification Approach

Several studies in the literature have integrated lung segmentation as a preprocessing step before classification, to confine the model′s learning to diagnostically meaningful regions. Alharbi and Mahmoud [18] developed a segmentation‐guided DL approach for pneumonia classification using CXRs. Firstly, the authors applied the lung segmentation to remove the irrelevant background interference using the BoxENet model, trained on 800 and tested on 800 expert‐annotated images. An enhanced BoxENet architecture incorporating transfer learning was employed to perform the classification task. The approach was evaluated on four public datasets comprising approximately 7700 CXR images (4000 normal and 3700 pneumonia images). The model achieved 97.40% accuracy on full CXR images and 95.40% when using segmented images for binary classification. Nimalsiri et al. [19] developed the CXLSeg dataset, encompassing 243,324 frontal chest x‐ray images. To perform the segmentation task, the authors analyze different U‐Net–based variants: Among them, a spatial attention–based U‐Net (SA‐UNet) architecture was trained and evaluated on the datasets SH, MC, and V7 labs, achieving good performance in terms of IoU (91.97%) and dice coefficient (96.80%). Furthermore, to examine the impact of segmentation on a multilabel classification task, the study used several CNNs, including ResNet50, DenseNet121, and InceptionV3. The results indicate that using segmented inputs from CXLSeg improved performance over MIMIC‐CXR images without segmentation. Building on the segmentation‐based classification approaches, Kumarasinghe et al. [20] modified the standard U‐Net architecture by incorporating residual convolutional blocks to improve the feature learning. Trained on V7‐labs COVID‐19 chest x‐ray dataset with the ground truth, the segmentation model attained a dice score of 96.55% and IoU of 93.53%. The segmented images were fed as input to train the ensemble of different CNN backbones, resulting in 99.36% accuracy and 99.39% F1‐score. Similarly, Yadav and Singhai [21] introduced a hybrid approach that first isolates the lung region from CXR images and then performs multiclass disease classification. To perform the segmentation task, the lung fields were isolated using a modified U‐Net model, the deep atrous attention U‐Net (DAA‐UNet), which achieved 98.12% accuracy and improved overlap metrics. After the segmentation task, both the original and segmented images were evaluated using multiple transfer learning–based architectures, including ChexNet, DenseNet201, ResNet101, and InceptionV3. Among them, DenseNet201 proved to be the most effective classifier, achieving an overall accuracy of 96.87% and balanced precision and recall. A recent review by Breesam et al. [22] highlighted that medical imaging tasks such as segmentation and classification play a vital role in isolating the ROI and assisting clinicians in achieving accurate diagnosis. The study explores the application of AI, particularly CNN‐based models, in analyzing various medical imaging modalities to delineate the ROI and identify pathological areas. Despite significant advancements, concerns persist regarding data availability, generalizability, and model interpretability for real‐world clinical implementation.

Overall, the evolution of LFS has progressed from traditional image processing methods to advanced DL frameworks, including attention‐guided networks, generative models, and vision–language integration. These innovations not only reflect the foundational significance of classical approaches but also demonstrate how they have enabled robust, annotation‐efficient, and clinically scalable segmentation solutions.

## 3. Proposed Method

The proposed method is divided into two stages:

Stage 1: Extraction and pruning of structural features based on Canny [23] and LoG [24] edge detection algorithms, followed by morphological processing to extract the significant features representing lung fields.
Stage 2: Here, the extracted features are combined with the original chest x‐ray images to produce multiple multichannel input configurations, which are then used to train and evaluate multiple DL‐based segmentation models for robust and generalizable LFS across diverse CXR datasets.

### 3.1. Stage 1

The input to this stage is a grayscale CXR image that is scaled to have *N* = 1024 columns while preserving the aspect ratio of the original image. To produce the desired outputs, the input image undergoes a series of processing steps, as outlined in the sections below:

#### 3.1.1. Blob Generation and Boundary Detection

The process begins by detecting the main body region (blob) to distinguish the foreground from the background in the input chest x‐ray image. To enhance the darker regions, a logarithmic transformation is initially applied to the input image *I*_*o*,_ after which contrast stretching is performed, producing the output image *I*_*p*_, as depicted in Equation (1).

(1) Ip=contrast_stretch logIo+ϵ 

Equation (2) determines the value of *ϵ* by computing the average local standard deviation for each pixel within an *m* × *n* neighborhood *S*_*x* *y*_. The size for the neighborhood is taken as *m* = 0.5∗*M* and *n* = 0.5∗*N*, where *M* × *N* represents the dimensions of the input image *I*_*o*_. The averaged value is scaled by the maximum intensity present in the original image *I*_*o*_.

(2) ϵ=1/MN∑x=0M∑y=0N∑p∈Sxyp−p¯2/mnmaxIo

**Figure 1:**
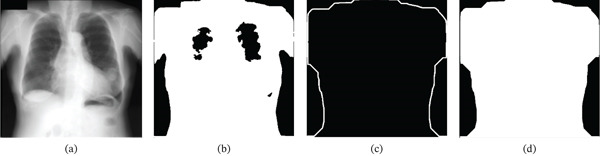
Visual representation of the blob generation and boundary detection process: (a) input Image  *I*_*o*_. (b) Binarized raw blob image *I*_*r*_. (c) Refined blob image, *I*_*b*_. (d) Blob mask boundary *M*_*b*_.

The resulting image *I*_*p*_ is thresholded at a value *T*_*b*,_ which is set to the local minimum of the histogram of *I*_*p*_ in the intensity range of [130–170] as used by [25]. This thresholding results in the binary image *I*_*r*_ as illustrated in Figure 1b.

Often, CXR images include artifacts and labels generated by overexposures. To address this issue, the blob image *I*_*r*_ of Figure 1b is morphologically processed by using pruning, isolated pixel cleaning, and closing. The structuring element used to perform closing involves the size *n* = min(M, N) × c, where *c* is a constant equal to 0.1. After processing, hole‐filling is applied, and the most prominent connected region is retained to represent the body blob *I*_*b*_ as shown in Figure 1c.

Furthermore, the boundary of the blob mask is extracted and morphologically dilated by a 3 × 3 structuring element of all ones to generate the boundary as illustrated in Figure 1d. The blob mask image *I*_*b*_ and the boundary mask *M*_*b*_ are used later to prune the edges from an edge‐detected image in the later section.

#### 3.1.2. LoG Edge Detection and Pruning

In this step, LoG filter is applied to detect edges from an image generated after processing the input image  *I*_*o*_ by performing the following steps.

1. To enhance the image contrast, “contrast‐limited adaptive histogram equalization (CLAHE),” as described in [26], is applied, using a tile grid size of 5 × 5, a clip‐limit threshold of 0.001, a “uniform” distribution, the number of bins to 256.
2. A 3 × 3 median filter is applied to remove the noise.
3. A 3 × 3 − min filter is applied to enhance lung field extraction, as it usually represents dark regions.

**Figure 2:**
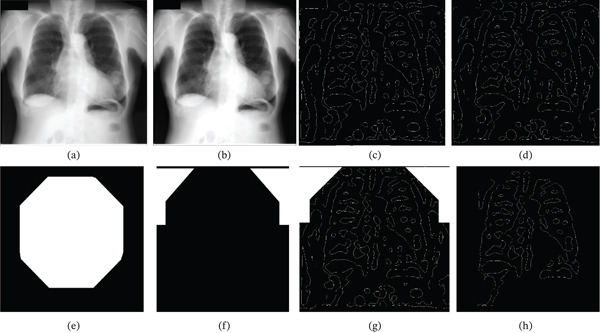
Representation of LoG edge detection and pruning. (a) Processed image  *I*_*c*_. (b) Contrast‐enhanced image  *I*_*d*_. (c) LoG edge image  *I*_*L*_, (d) image after boundary mask subtraction, and *L*_1_.(e) Mask after closing and erosion,  *M*_*R*_. (f) Dilated and complemented mask,  *M*_*S*_. (g) Image after removing shoulder edges using  *M*_*S*_ from *L*_1_, resulting in *I*_*R*_. (h) Reconstructed lung fields  *L*_*F*_ obtained from *I*_*R*_ using the  *M*_*R*_.

The resulting image, referred to as  *I*_*c*_, as shown in Figure 2a is a contrast stretched between [0.05–0.95] to remove low‐intensity noise and high‐intensity saturation. Further, CLAHE is applied by setting the “tile grid size to 5 × 5, the distribution to *‘*Rayleigh*,’* number of bins to 256, and the clip‐limit threshold to 0.005 and the alpha to 0.7”, resulting in image  *I*_*d*_ as shown in Figure 2b. This process is then followed by applying a 3 × 3 LoG edge detection on the resulting image with variance *σ* ^2^ = min(*M*, *N*) × *ϵ* × *c*. Here, *ϵ* is calculated using Equation (2) on the image of Figure 2a, and *c* is a constant equal to 0.6. The filter size is *n* ×*n*, where *n* = ceil(*σ* ^2^ × 3) × 2 + 1. It should be noted that the parameters used in the proposed heuristic stage are not fixed constants. Rather, they are derived from the input image properties themselves. In particular, the variance parameter used in the edge detection process is computed as a function of the image dimensions *M* and *N*. This allows the edge detection to automatically adjust according to the spatial resolution of the input CXR image. This adaptive approach ensures that structural features remain robust across images acquired under different imaging conditions and resolutions.

The resulting LoG edge image, referred to as  *I*_*L*_, is shown in Figure 2c. It can be seen that the LoG edge features significantly consist of the representation of lung fields as closed boundaries. However, it also consists of numerous other closed edges that do not represent RoIs. To remove and disconnect some of the unwanted closed edges, the boundary  *M*_*b*_ of Figure 1d is subtracted from the LoG edge image  *I*_*L*_ of Figure 2b, resulting in the image  *L*_1_ shown in Figure 2d and Equation (3).

(3) L1=IL−ILMb

In order to extract only the edges representing the lung fields, the proposed approach constructs two masks,  *M*_*R*_ and  *M*_*S*_, as shown in Figure 2e,f, respectively. Figure 2e represents the  *M*_*R*_ mask generated from image *I*_*b*_ of Figure 1c by applying closing using a disk structuring element of all ones of size *n* = min(*M*, *N*) × *c*, where *c* is a constant equal to 0.7. This is followed by erosion operation with a disk structuring element of all ones of size calculated using Equation (4), where *c* is a constant equal to 6. This mask is expected to encompass the ribcage area of the CXR image.

(4) Sen=round min M,N×ϵ×c

The mask  *M*_*S*_ of Figure 2f is constructed by complementing the upper 50% of the mask  *M*_*R*_ of Figure 2e and then dilating by a disk structuring element of all ones with size given by Equation (4) by setting *c* set equal to 0.05. The mask  *M*_*S*_ of Figure 2f is used to remove the edges representing the shoulders in the LoG edge image *L*_1_of Figure 2d by subtracting all the reconstructions of image *L*_1_ of Figure 2d using mask  *M*_*S*_ of Figure 2 f from *L*_1_ as shown in Figure 2g and Equation (5).

(5) IR=L1−reconstruct L1,MS

The  *M*_*R*_ mask of Figure 2e is then used to reconstruct the RoIs from the resulting image *I*_*R*_, which gives a better representation of the lung fields referred to as *L*_*F*_ as illustrated in Figure 2h.

##### 3.1.2.1. Morphological Processing and ROI Detection.

**Figure 3:**
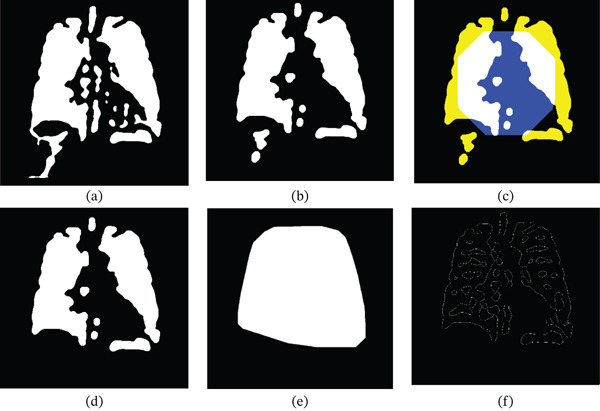
Illustration of Morphological Processing and ROI Detection. (a) Enhanced image  *S*_1_ from the convex hull of image *L*_*F*_. (b) Morphologically processed image  *S*_2_ obtained through opening operations on  *S*_1_.(c) Reconstruction of  *S*_2_ using  *M*_*R*_. (d) Reconstructed image  *S*_3_. (e) Convex hull constructed from  *S*_3_. (f) Final image reconstructed using  *C*_1_, yielding *I*_*F*_.

This subprocess involves further pruning of the initial RoIs image *L*_*F*_ extracted as shown in Figure 2h. The first step here is to generate the convex hull of the *L*_*F*_ image and use that to reconstruct the edge features from image  *L*_1_ of Figure 2d. The resulting image is then dilated, hole‐filled, and closed to generate image  *S*_1_ as illustrated in Figure 3a. The structuring element used for dilating and closing is generated using Equation (3) with *c* set equal to 1.5. The resulting image is further morphologically opened using the same structuring element to yield the image  *S*_2_ as shown in Figure 3b. Now, in order to extract the significant representation of the lung fields, the proposed method involves using an eroded version of the mask  *M*_*R*_ of Figure 2e, for which the disk structuring element is generated by using Equation (3) with *c* set equal to 3. This mask is then used to reconstruct image  *S*_2_ of Figure 3b as shown in Figure 3c, which yields image  *S*_3_ of Figure 3d. Now, the process proceeds with constructing the convex hull of the image  *S*_3_ of Figure 3d to get the image  *C*_1_ of Figure 3e. The mask  *C*_1_ is further eroded by a disk structuring element generated using Equation (3) with *c* set equal to 2 and used to reconstruct all the edge features from image *L*_*F*_ of Figure 2h. This operation, as shown in Equation (6), yields the image *I*_*F*_ of Figure 3f.

(6) IF=reconstructLF,C1

It is clear that by using this stage of the proposed process, the lung representation of the extracted LoG edge features is highly significant, as they include the complete representation of the lung fields. However, the image *I*_*F*_ of Figure 3f still consists of certain contours that do not represent portions of lung fields but instead represent the region of the spine and other parts in the upper abdomen. These edge contours are removed using the following stages.

##### 3.1.2.2. Medial Axis Detection and Spine Removal.

**Figure 4:**
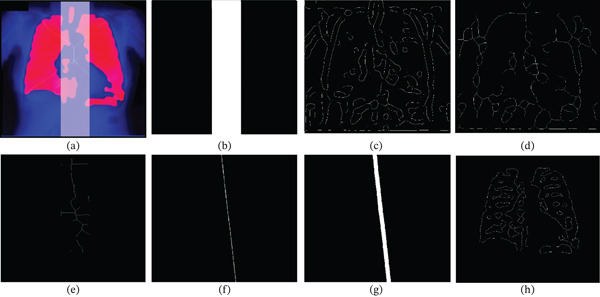
Illustration of medial axis detection and spine removal. (a) Vertical band overlapped with RoI. (b) Isolated vertical band mask  *B*_1_ representing the spine. (c) Edge image  *X*_0_ from CLAHE enhancement and Canny edge detection. (d) Skeletonized image  *X*_1_ from  *X*_0_.. (e) refined spine representation  *X*_2_ after hole‐filling and skeletonization. (f) Representation of the smooth spline curve,  *X*_3_. (g) Thickened spline,  *X*_*m*_. (h) RoI with spine features removed, (LoG representation) labeled as *I*_LX_.

The primary objective of this stage is to remove any edge features that do not represent lung fields but instead are representations of the spine. In order to do this, initially, a crude mask representation of the spine area is generated by constructing a vertical band at the centroid of the convex hull of image  *C*_1_ of Figure 3e. The thickness of the band is taken as twice the 15% of the width of the region shown in image  *C*_1_ of Figure 3e. This thickness value is not fixed but is proportionally derived from the actual width of the detected region, making it adaptive to the size of structures present in each individual chest x‐ray image. The band thus constructed is illustrated as mask  *B*_1_ in Figure 4a,b.

Next, the input image  *I*_*c*_, of Figure 2a undergoes CLAHE enhanced with a 5×5 tile grid, 256 histogram bins, the distribution to “Rayleigh,” and the clip‐limit threshold to 1 and alpha value to *0.2*, after which contrast stretching in the range [0.4–1] is applied to enhance the higher intensities representing the bone tissue. Next, the canny edge detection algorithm [23] is applied with thresholds  *T*_1_ = *ϵ*  and *T*_2_ = 2. Furthermore, the contrast stretching and threshold parameters used during edge extraction are determined based on the normalized intensity range of the input image, rather than predefined fixed values, allowing the enhancement to adapt to varying brightness levels and contrast across different chest x‐ray datasets. The variance for the edge detection is taken as *σ* ^2^ = min (*M*, *N*) × *ϵ* × *c*. Here, *ϵ* is calculated using Equation (2) on the  *I*_*c*_ image of Figure 2a, and *c* is a constant equal to 0.67. The size of the filter is *n* ×*n*, where  ^"^ *n* = ceil(*σ* ^2^ × 3) × 2 + 1^"^. The resulting edge image  *X*_0_ shown in Figure 4c is closed with a disk structuring element of all ones with size given by Equation (3) by setting *c* set equal to 1. The resulting image is skeletonized, resulting in image  *X*_1_ as shown in Figure 4d.

Now, to extract the spine representation, the image  *X*_1_ of Figure 4d is further hole‐filled, skeletonized, and ANDed with the masks  *B*_1_ and  *C*_1_ of Figures 3e and Figures 4b, respectively, to get the image  *X*_2_ of Figure 4e. The edges thus extracted are a close representation of the orientation and position of the spine in the chest radiographs. The image  *X*_2_ of Figure 4e is used to fit a smooth spline curve along the *y*‐axis and thickened to represent the spine more closely, as shown in images  *X*_3_ and  *X*_*m*_ of Figure 4f,g, respectively. The image  *X*_*m*_ of Figure 4g is then used to remove all the reconstructions of image *I*_*F*_ of Figure 3f from  *I*_*F*_ as shown in Equation (7), yielding the image *I*_*L* *X*_ of Figure 4h.

(7) ILX=IF−reconstructIF,Xm

It is clear that the image *I*_*L* *X*_ of Figure 4h is a closer representation of the lung fields with significant spine removed from the same.

#### 3.1.3. Extracting the Canny Edge Features

In order to refine the RoIs to closely represent the lung fields, the proposed approach further utilizes the Canny edge detection algorithm on two different intensity ranges of the input image *I*_*d*_ of Figure 2b.

**Figure 5:**
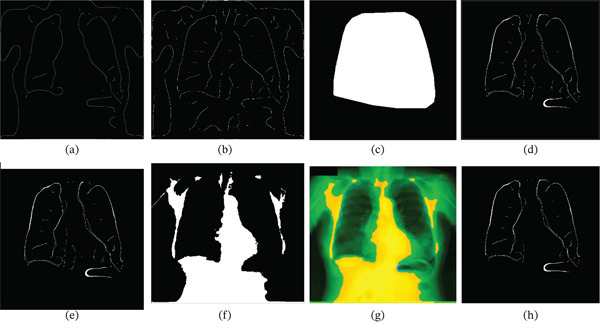
Illustration of extracting the canny edge features. (a) Canny image  *C*_1_ from input image *I*_*d*_. (b) Canny image  *C*_2_ generated with thresholds *T*_1_ = *ϵ* and *T*_2_ = 2. (c) Convex hull  *X*_*L*_ used to extract RoIs from  *C*_*F*_. (d) Reconstructed image  *C*_*F*_ from the OR operation between  *C*_1_ and  *C*_2_ using  *X*_*L*_. (e) Image after spine feature removal,  *C*_*P*_. (f) Saturated binary mask  *S*_*M*_. (g) Removal of spine edges from  *C*_*P*_ using  *S*_*M*_. (h) Mask *I*_CN_ representing the closest lung field boundary.

In the first case, the canny edge detected in image  *C*_1_ of Figure 5a is generated by using the canny thresholds *T*_1_ = (*ϵ* × 10) − 0.4 and *T*_2_ = 2 on the input image that is contrast stretched between [0.4–0.73]. The variance for the edge detection is taken as *σ* ^2^ = min(*M*, *N*) × *ϵ*. Here, *ϵ* is calculated using Equation (2) on the image *I*_*d*_ of Figure 2b. Similar to the LoG stage this variance is computed from the input images dimensions, ensuring consistent resolution‐ aware edge detections across different imaging conditions. The size of the filter is *n* ×*n*, where*n* = ceil (*σ* ^2^ × 3) × 2 + 1. In the second case, another canny edge detected in image  *C*_2_ of Figure 5b is generated by using thresholds *T*_1_ = *ϵ* and *T*_2_ = 2 on the image *I*_*d*_ of Figure 2b that is contrast stretched between [0.4–0.73]. The variance for the edge detection is taken as *σ* ^2^ = min(*M*, *N*) × *ϵ* × *c*. Here, *ϵ* is calculated using Equation (2), and *c* is a constant equal to 0.7, and the size of the filter is *n* ×*n*, where *n* = ceil(*σ* ^2^ × 3) × 2 + 1. Although a scaling constant of 0.7 is applied here, the base variance remains image‐driven through Equation (2), preserving the adaptive nature of the detection process. The two contrast ranges are used here to ensure that Canny edge images significantly demarcate lung RoIs in case of low variance images or images with pneumonia, etc. To extract the lung field edges from the Canny features, the convex hull  *X*_*L*_ of image *I*_*L* *X*_of Figure 4h, as shown in Figure 5c, is used to reconstruct all the edge features from the OR of Figure 5a,b, as shown in Equation (8).

(8) CF=reconstructC1C2,XL

The resulting image  *C*_*F*_ is shown in Figure 5d. Now the process of spine features removal, as discussed in Section B.2, is also applied here, where in the image  *X*_*m*_ of Figure 4g is used to reconstruct all edge features from image  *C*_*F*_ of Figure 5d, which are then removed from the same to get the image  *C*_*P*_ of Figure 5e.To further prune the canny edges, a saturated mask primarily representing the spine, ribcage boundary, and abdomen area is constructed by applying CLAHE on the input image *I*_*d*_ with clip Limit 1, distribution “Rayleigh*,”* and alpha value equal to 0.7, number of bins 256, followed by contrast stretching within the range [0.9–1] if 2 × mean(*I*_*d*_)/256 > 1; else in the range [2 × mean(*I*_*d*_) − 1].

The resulting image is further binarized to get the mask  *S*_*M*_ of Figure 5f. This mask is used to reconstruct all the edges from image  *C*_*P*_ of Figure 5e, which represents the spine which is then removed from the same as shown in Figure 5g,h. The image *I*_*C* *N*_ of Figure 5h is the closest representation of the lung field boundary so far. However, it still consists of some representations from the upper that are not the parts of the lungs.

##### 3.1.3.1. Left and Right Lung Separation and Pruning.

**Figure 6:**
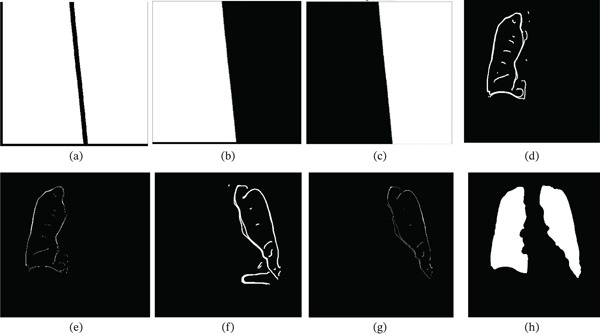
Illustration of left and right lung separation and pruning. (a) Complemented spine mask *X*_*M*_. (b) Left lung regions L_S_ extracted from *X*_*M*_. (c) Right lung region R_S_ extracted from *X*_*M*_. (d) Enhanced left lung Left_C_. (e) Pruned left lung *L*_*p*_. (f) Enhanced right lung Right_*C*_. (g) Pruned right lung representation *R*_*p*_. (h) Minimal RoI representation, *X*_*M*_.

In this section, the proposed method separates the left and right lung Canny features so as to further prune and refine the lung representation. To do this, the spine mask  *X*_*M*_ of Figure 4g is complemented, and the two connected components representing the left  *L*_*S*_ and the right  *R*_*S*_ segments, as shown in Figure 6b,c, respectively, are extracted. These two segment masks are used to extract the left and the right lung representation from the Canny edge image *I*_*C* *N*_ of Figure 5h using reconstruction as shown in Equations (9) and (10).

(9) LeftL=reconstruct ICN,LS

(10) RightL=reconstruct ICN,RS

These reconstructions are further morphologically enhanced using dilation with an *n* × *n* disk structuring element of ones, where *n* is calculated using Equation (11), to yield image Left_*C*_ of Figure 6d and image Right_*C*_ of Figure 6f, respectively. In Equation (11), ht is the height of the convex hull  *X*_*L*_ of Figure 5c and *c* is a constant set to 0.006.

(11) n=round ht×c

Furthermore, the left and right lung representations thus obtained are further pruned by preserving only the largest two connected components from each of the Left_*C*_ and Right_*C*_ RoIs, whose convex hulls are used to reconstruct image *I*_*L* *X*_ of Figure 4h to yield the left Canny representation *L*_*p*_of Figure 6e and right canny representation *R*_*p*_of Figure 6g. These two representations are further morphologically processed by performing closing and dilation with an *n* × *n* disk structuring element of ones, where *n* is calculated using Equation (11), with *ht* equal to the height of the convex hull  *X*_*L*_ of Figure 5c and *c* is a constant set to 0.005 and 0.03, respectively. Since *n* is derived from the convex hull of the detection lung region, the size of the structuring element scales proportionally with the anatomical dimensions of the lungs in each image. This adaptive formulation allows the morphological parameters to be determined based on the geometric properties of the detected lung regions rather than relying on fixed constants. The resultant processed regions are then subjected to hole filling to produce a combined OR result of Figure 6h, referred to as image *C*_*M*_. Image *C*_*M*_ is the minimal representation of the lung fields. These minimal representations are further enhanced by combining them with the LoG representations as extracted in image *I*_*L* *X*_ of Figure 4h, which is illustrated in the following section.

##### 3.1.3.2. Combining LoG and CANNY ROIs.

**Figure 7:**
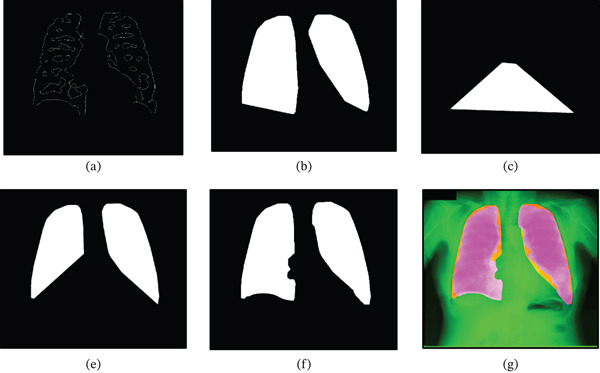
Illustration of combining LoG and Canny ROIs. (a) Reconstructed image  *I*_*X*_, from *I*_LX_and minimal RoI *C*_*M*_.(b) Convex hull M_L_, from *I*_*X*_. (c) Polygon *T*_1_ representing abdominal features. (d) Resulting image *P*_1_ after subtracting *T*_1_from *M*_*L*_. (e) Final combined RoI *L*_*F*_ obtained by ORing *P*_1_with *C*_*M*_. (f) Final segmented lung fields *L*_*F*_ overlapped on the original image.

In this final stage, the proposed method combines the LoG representation *I*_*L* *X*_ of Figure 4 h and the minimal representation of lung fields *C*_*M*_ from Figure 6h as follows:

Initially, the LoG representation *I*_*L* *X*_ of Figure 4 h is reconstructed by the minimal representation of the lung fields *C*_*M*_ as shown in Equation (12), resulting in image *I*_*X*_ of Figure 7a.

(12) IX=reconstructILX,CM

Next, the convex hull of image *I*_*X*_ of Figure 7a is determined as shown in image  *M*_*L*_ of Figure 7b. The bounding boxes of each of the lungs are determined and represented by [*T* *L*_*X*_,*T* *L*_*y*_,*B* *L*_*X*_, *B* *L*_*y*_] for the left lung and [*T* *R*_*X*_, *T* *R*_*y*_, *B* *R*_*X*_, *B* *R*_*y*_] for the right lung, respectively. To remove any abdominal representation in the LoG convex hull, a polygon *T*_1_ representing possible abdominal features, as shown in Figure 7c, is subtracted from image *M*_*L*_ as shown in Equation (13). This polygon is created from four points represented as follows:

BRx,TRy+BRy−TRy/2,TLx,TLy+BLy−TLy/2,BLx,BLyTRx,BRy.P1=ML−T1

The resulting image *P*_1_ shown in Figure 7d is then ORed with the minimal representation *C*_*M*_ of Figure 6h to generate the final combined representation as shown in Figure 7e and Equation (14). This final mask *L*_*F*_ of Figure 7e, representing the extracted lung fields, is overlapped with the original image for reference in Figure 7 h.

(14) LF=P1CM

### 3.2. Early Fusion of Multichannel Structural Feature for DL‐Based LFS

In this stage, the heuristic features extracted in Stage 1, namely two canny edge variants (cn_1_, cn_2_), a LoG, and a predicted mask (pm) generated from the heuristic approach without any ground truth supervision, are systematically integrated with the *I*_*o*_ to produce enriched input configurations. Each possible selection of channels is referred to as a channel combination *C*_*k*_ where *k* represents the combination index, as expressed in Equation (15), each combination *C*_*k*_ includes *I*_*o*_ as the base channel, along with a subset H_k_ of heuristic features:

(15) Ck=Io∪Hk,where Hk⊆H

where *H*_*k*_ denotes the subset of subset of the channels selected in the *k* ^th^ combination and *H* = {cn_1_, cn_2_, log, pm}. Since *I*_*o*_ is always active and each of the four channels independently has two possible states, the total number of distinct combinations is derived as *N*_comb_ = 2^|*H*|^ = 2^4^ = 16. Therefore, *k* ranges from 1 to 16 possible channel combinations, covering all the possible structural configurations evaluated in this study. To construct the multichannel input tensor, all selected channels are loaded, resized to 256 × 256, and stacked together to form a five‐channel tensor *X* ∈ R^(*B*, *C*, *H*, *W*)^, where *B* is batch size, *C* = 5 denotes the number of input channels, and *H* = *W* = 256. Prior to being fed these configurations to the segmentation models, the input tensor *X* is passed through learnable channel mixer, and simultaneously a binary selection mask m ∈ {0, 1}^*c*^  is applied to retain the active channels and zero out inactive ones, as expressed in Equation (16).

(16) X~=X⊙m,m∈01,C,where⊙denotes the element wise multiplication

The resulting masked tensor $\tilde{\text{X}}$ is then projected through 1 × 1 convolution to produce an enriched three‐channel representation, as expressed in Equation (17).

(17) Xfused=Conv11xX~

**Figure 8:**
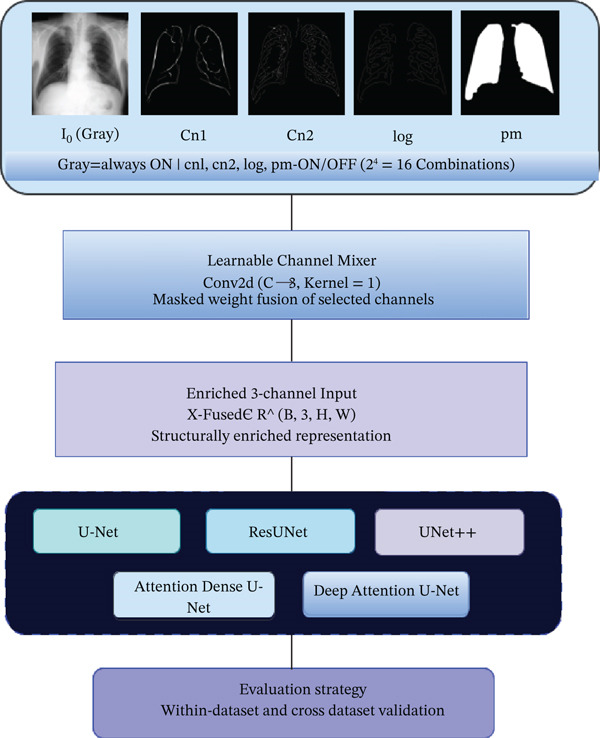
Workflow diagram for Stage 2.

The weight corresponding to the grayscale channel *I*_*o*_ is initialized to 1.0 to preserve the image information, whereas the remaining weights are learned adaptively during training, producing *X*_fused_ = ∈R^(*B*, 3, *H*, *W*)^. The resulting *X*_fused_ representation is subsequently fed as the input to the various segmentation model for training and evaluation using the early fusion strategy. The overall workflow of the Stage 2 for multichannel structural feature fusion and segmentation model training is illustrated in Figure 8.

## 4. Experimental Setup

### 4.1. Datasets

To evaluate the effectiveness of the proposed LFS approach, experiments were conducted on three publicly available benchmark CXR datasets: MC, United States, SH [27], and JSRT [28]. Table 1 summarizes the characteristics of the CXR datasets used in this study, including image counts, availability of ground truth masks, training‐testing splits, and image resolution.

**Table 1:** Overview of CXR datasets used in this study.

Dataset	No. of images	Number of ground truth masks	Training set	External test set	Cardinality
JSRT	247 PA CXRs	247	197	50	2048 × 2048
MC	138 PA CXRs	138	110	28	4020 × 4892 or 4892 × 4020
SH	662 PA CXRs	566	453	113	∼3000 × 3000

### 4.2. Segmentation Models

DL‐based encoder–decoder architectures have emerged as the standard approach for medical image segmentation tasks. U‐Net [6] stands out as a foundational model due to its symmetric downsampling and upsampling paths, which combine the high‐level semantic features with precise localization information. In this study, U‐Net along with its variants: Deep attention U‐Net [29], U‐Net++ [30], ResUNet [31], and attention dense U‐Net [32, 33], were evaluated to analyze the impact on the segmentation performance. All the models were trained using a standardized input resolution of 256 × 256 pixels.

### 4.3. Implementation Details

All experiments were conducted on a high‐performance computing server, configured with an NVIDIA A100‐SMX4‐40GB GPU with 40‐GB RAM along with 1007‐GB system memory, installed at our institution. The DL models were implemented pytorch framework (Version 1.13) with CUDA support along with the standard python‐based scientific computing libraries. The training and evaluation were developed in python using commonly used libraries for numerical computation, data processing, and visualization.

### 4.4. Training Strategy

All the CXR images were preprocessed by resizing to 256 × 256 and converted to grayscale, with the pixel intensities normalized to the [0,1] range to ensure consistency and improved training stability. The models were optimized using an Adam optimizer with a learning rate of 1 × 10^−4^, and BCEWithLogits loss serves as the loss function to handle the binary segmentation task effectively. Training was conducted for 50 epochs with a batch size of 4. Model evaluation was conducted under two validation protocols: five‐fold cross‐validation to assess intradataset reliability, and cross‐dataset validation to examine interdataset generalizability, as detailed in the subsequent section.

### 4.5. Segmentation Evaluation Metrics

To evaluate how well the models segmented the lung regions, several key metrics are used that measure how closely the prediction masks are matched with the expert‐annotated reference masks. To measure overlap and overall segmentation quality, we used the DSC and Jaccard score (IoU), both of which are particularly useful for medical image segmentation. In addition, metrics such as accuracy, precision, and recall were computed to evaluate the segmentation performance in terms of labelling individual pixels.

## 5. Results

### 5.1. Validation Strategy

To rigorously evaluate the segmentation robustness and generalizability, both intradataset and cross‐dataset validation protocols were adopted. To ensure fair and eliminate evaluation bias, separate hold‐out test subsets were defined for each dataset prior to model training. For intradataset evaluation, 20% of each dataset was reserved exclusively for testing and evaluation purposes, and the remaining 80% was used for training. The training set was subjected to five‐fold cross‐validation to ensure robust internal validation in order to avoid the risk of overfitting. In each iteration, four folds were used for model training, whereas the remaining fold served as a validation for performance monitoring. Following the cross‐validation, the model was evaluated on the separate held‐out test set to obtain final performance metrics results.

To further assess domain generalizability, cross‐dataset validation was conducted, where the model was trained exclusively on one dataset and evaluated on the reserved hold‐out subset of a different dataset, without fine‐tuning, which ensures consistency and prevents data leakage.

### 5.2. Ablation Study

To better understand and analyze the impact of each structural channel on lung segmentation performance, a thorough ablation study was conducted by considering all the unique channel combinations generated in Stage 2 across all the considered models under both intradataset and interdataset validation settings. For each configuration *C*_*k*__,_ the segmentation models were trained with the same experimental setting to ensure a fair and unbiased comparison across all combinations. This systematic analysis makes it possible to clearly observe how the individual structural channels, from single input to more complex multi‐channel combinations, influence segmentation performance and to identify the most consistent channel combination across all the datasets used in this study.

Given the large number of experimental configurations involved, the complete quantitative findings for all combinations across all the models and datasets are provided in the Supporting Information (Tables S1, S2, S3, S4, S5, S6, S7, S8, S9, S10, S11, S12, S13, S14, S15, S16, S17, S18, S19, S20, S21, S22, S23, S24, S25, S26, S27, S28, S29, S30, S31, S32, S33, S34, S35, S36, S37, S38, S39, S40, S41, S42, S43, S44, and S45), covering both validation strategies with all five metrics reported for every configuration. Among all the evaluated configurations and architectures, the most consistent and highest performing configurations are reported in the result section, where the best performing model is further compared against SOTA methods to demonstrate the effectiveness of the proposed approach.

### 5.3. Segmentation Results

This section presents the segmentation performance achieved by the proposed multichannel feature fusion approach under both within and cross‐dataset validation settings. The result section is organized to first report the within‐dataset performance across the JSRT, MC, and SH datasets, followed by the cross‐dataset validation results to assess the generalizability of the proposed approach across unseen imaging domains.

#### 5.3.1. Evaluation on Interbenchmark Datasets

Across the three datasets, configurations that included structural edge features (cn2 and log) consistently outperformed the baseline (*I*_*o*_) configuration. Deep attention U‐Net achieved consistent results when trained on channel combination I_o__cn2_log_pm. As summarized in Table 2, dice gains ranged from +0.0011 to +0.0033, whereas IoU improvements remained within +0.0009 to +0.0021 across the three datasets, maintaining balanced dice and Jaccard scores.

**Table 2:** Within dataset segmentation performance of deep attention U‐Net using baseline and consistent channel combination (*I*_o__cn2_log_pm).

Dataset	Baseline dice	Baseline IoU	Channel dice	Channel IoU	*Δ* *d* *i* *c* *e*	*Δ* *I* *o* *U*
JSRT	0.9803	0.9615	**0.9815**	**0.9624**	+0.0013	+0.0009
MC	0.9771	0.9556	**0.9782**	**0.9577**	+0.0011	+0.0021
SH	0.9547	0.9190	**0.9581**	**0.9203**	+0.0034	+0.0013

*Note:* The bolded values highlight the best segmentation results obtained using the proposed channel combination approach. These values correspond to the channel dice and Channel IoU metrics, which consistently show improved performance compared to the baseline (*I*_o_) across all datasets. Notably, the deep attention U‐Net model achieves the highest performance among all models evaluated in the within‐dataset setting.

Similarly, U‐Net ++ showed modest but consistent performance when integrating *I*_o__cn2, suggesting that shallow skip connections benefit from enhanced boundary cues. As shown in Table 3, the most noticeable improvement was observed on MC dataset, where IoU increased by +0.0041.

**Table 3:** Within‐dataset segmentation performance of U‐Net++ using baseline and consistent channel combination (*I*_o__cn2).

Dataset	Baseline dice	Baseline IoU	Channel dice	Channel IoU	*Δ* *d* *i* *c* *e*	*Δ* *I* *o* *U*
JSRT	0.9799	0.9607	**0.9810**	**0.9614**	+0.0011	+0.0007
MC	0.9751	0.9520	**0.9775**	**0.9561**	+0.0024	+0.0041
SH	0.9581	0.9201	**0.9586**	**0.9211**	+0.0005	+0.0010

*Note:* Among the 16 channel combinations evaluated, the *I*_o__cn2 combination consistently performs better with the U‐Net++ model across all three datasets, compared with the baseline that considers only the original images (*I*_o_). The bolded values highlight these improved results, corresponding to the channel dice and IoU metrics.

In contrast, the standard U‐Net exhibited the highest gain when integrated with most of channel combinations in terms of dice and Jaccard. As presented in Table 4, dice increased by +0.421 and IoU by +0.0739 on the JSRT dataset. Comparable improvements were observed in MC and SH datasets, with dice gains exceeding +0.0199 and IoU improvements exceeding +0.0317. These findings indicate that simple encoder–decoder structure of U‐Net benefits more from edge‐assisted integration.

**Table 4:** Within‐dataset segmentation performance of standard U‐Net using baseline and consistent channel combination (*I*_o__cn2_log_pm).

Dataset	Baseline dice	Baseline IoU	Channel dice	Channel IoU	*Δ* *d* *i* *c* *e*	*Δ* *I* *o* *U*
JSRT	0.9139	0.8420	**0.9560**	**0.9159**	+0.0421	+0.0739
MC	0.9157	0.8478	**0.9356**	**0.8795**	+0.0199	+0.0317
SH	0.9236	0.8600	**0.9450**	**0.8967**	+0.0214	+0.0367

*Note:* The bolded values represent the segmentation performance achieved using the proposed channel combination with the standard U‐Net model. Compared with the baseline (*I*_o_), the integration of enhanced channels (*I*_o__cn2_log_pm) results in notable improvements in both dice and IoU scores across all datasets. These values highlight the significant performance gains of the proposed approach, further supported by the corresponding ΔDice and ΔIoU metrics.

**Figure 9:**
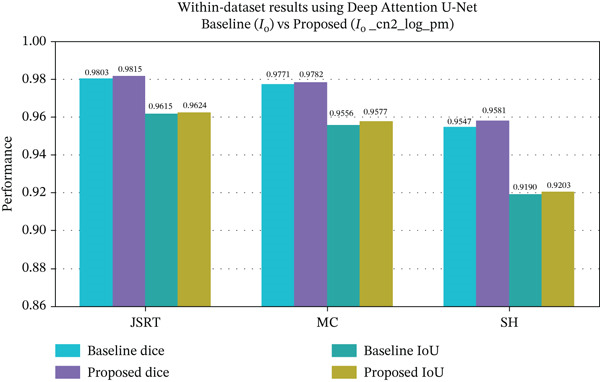
Within‐dataset evaluation of deep attention U‐Net, comparing the baseline configuration (*I*_o_) with the proposed configuration across the JSRT, MC, and SH datasets.

Figure 9, presents the within‐dataset evaluation results of the best performing model, deep attention U‐Net using the baseline and the proposed channel combination across the three datasets. The comprehensive quantitative results for all channel combinations and architectures, including all the performance metrics values, are provided in the Supporting Information (Tables S1, S2, S3, S4, S5, S6, S7, S8, S9, S10, S11, S12, S13, S14, and S15).

#### 5.3.2. Cross‐Dataset Evaluation

To rigorously evaluate segmentation generalizability, cross‐dataset validation was performed to address the real‐world clinical scenarios where data characteristics vary significantly due to differences in imaging protocols, equipment, and patient demographics. This approach reveals how well models handle the unseen data from diverse distributions, offering insights into their practical applicability. The quantitative results of cross‐dataset evaluation are summarized in Tables 5, 6 and 7.

**Table 5:** Cross‐dataset segmentation performance of Deep Attention U‐Net using baseline and consistent channel combination (*I*_o__cn2_log_pm).

Dataset	Baseline dice	Baseline IoU	Channel dice	Channel IoU	*Δ* *d* *i* *c* *e*	*Δ* *I* *o* *U*
JSRT →MC	0.9331	0.8849	**0.9462**	**0.9021**	+0.0131	+0.0172
MC → JSRT	0.9621	0.9275	**0.9626**	**0.9280**	+0.0005	+0.0005
JSRT →SH	0.9322	0.8785	**0.9454**	**0.8992**	+0.0132	+0.0207
SH → JSRT	0.9576	0.9195	**0.9598**	**0.9224**	+0.0022	+0.0029
MC → SH	0.9369	0.8833	**0.9422**	**0.8928**	+0.0053	+0.0095
SH → MC	0.9356	0.8833	**0.9556**	**0.9167**	+0.0200	+0.0334

*Note:* The bolded values highlight the segmentation performance achieved using the proposed consistent channel combination in a cross‐dataset setting with the deep attention U‐Net model. These results highlight that the deep attention U‐Net is the best‐performing model among the evaluated approaches, consistently outperforming the baseline (*I*_o_) in terms of dice and IoU, indicating enhanced generalization capability.

**Table 6:** Cross‐dataset segmentation performance of U‐Net++ using baseline and consistent channel combination (*I*_o__cn2).

Dataset	Baseline dice	Baseline IoU	Channel dice	Channel IoU	*Δ* *d* *i* *c* *e*	*Δ* *I* *o* *U*
JSRT →MC	0.9312	0.8825	**0.9534**	**0.9132**	+0.0222	+0.0307
MC → JSRT	0.9551	0.9147	**0.9637**	**0.9302**	+0.0086	+0.0155
JSRT →SH	0.8409	0.7322	**0.9477**	**0.9029**	+0.1068	+0.1707
SH → JSRT	0.8761	0.7981	**0.9524**	**0.9102**	+0.0763	+0.1121
MC → SH	0.9338	0.8787	**0.9378**	**0.8865**	+0.0040	+0.0078
SH → MC	0.7303	0.6060	**0.9327**	**0.8783**	+0.2024	+0.2723

*Note:* The bolded values represent the segmentation results obtained using the proposed channel combination in a cross‐dataset setting with the U‐Net++ model. These values correspond to the channel dice and IoU metrics, which consistently outperform the baseline across all cross‐dataset scenarios, reflecting the model′s enhanced generalization capability.

**Table 7:** Cross dataset segmentation performance of standard U‐Net using baseline and consistent channel combination (*I*_*o*__cn2_log_pm).

Dataset	Baseline dice	Baseline IoU	Channel dice	Channel IoU	*Δ* *d* *i* *c* *e*	*Δ* *I* *o* *U*
JSRT →MC	0.8420	0.7350	**0.8969**	**0.8214**	+0.0549	+0.0863
MC → JSRT	0.7027	0.5493	**0.9316**	**0.8727**	+0.2288	+0.3233
JSRT →SH	0.5273	0.3830	**0.8931**	**0.8106**	+0.3657	+0.4275
SH → JSRT	0.5925	0.4291	**0.9347**	**0.8783**	+0.3422	+0.4492
MC → SH	0.2211	0.1490	**0.9070**	**0.8320**	+0.6859	+0.6829
SH → MC	0.4778	0.3208	**0.8922**	**0.8275**	+0.4143	+0.5066

*Note:* The bolded values show the segmentation results achieved using the proposed channel combination in a cross‐dataset setting with the standard U‐Net model. These values correspond to the channel dice and IoU metrics, and they consistently perform better than the baseline across all cross‐dataset scenarios. It is also observed that the standard U‐Net achieves comparatively larger performance gains, indicating its effectiveness and improved generalization ability.

For deep attention U‐Net (Table 5), the proposed consistent channel combination (*I*_*o*___*c* *n*2__log__*p* *m*) yielded moderate performance across all the transfer scenarios. Dice gains ranged from +0.0005 to +0.0200 over baseline, with IoU improvements reaching up to +0.0334 (SH → MC). This indicates that attention‐based architectures maintain relatively stable cross‐domain stability, with the addition of edge‐based features.

The cross‐dataset performance of the U‐Net ++ is presented in Table 6. Although U‐Net ++ showed relatively strong baseline performance, the integration of consistent channel combination _(*I*_o__cn2) further improved the overlap‐based metrics across all source‐target pairs. A notable improvement was observed in JSRT→SH, where IoU increased by 0.1707. Even more substantial gains were recorded in SH → MC, where dice improved by 0.2024 and IoU increased by 0.2723. The results suggests that multiscale skip refinement in U‐Net ++ benefits from the channel integration under severe domain conditions.

Finally, Table 7 details the cross‐dataset validation results of standard U‐Net architecture, which demonstrates the pronounced gain in terms of dice and IoU when integrated with almost all the channel combinations compared with the baseline configuration.

**Figure 10:**
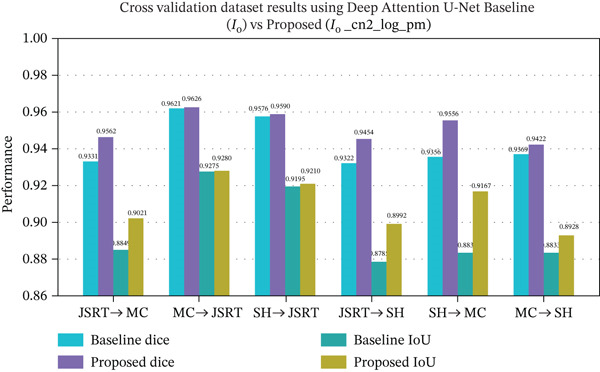
Cross‐dataset validation results of the best performing model, deep attention U‐Net, comparing the baseline configuration with the proposed channel combination across all six cross‐dataset combinations.

Overall, the cross‐dataset experiments reveal that the proposed multiscale edge‐assisted features substantially enhance the segmentation robustness across all the metrics. Figure 10 presents the cross‐dataset validation results of the best performing model, deep attention U‐Net, comparing the baseline configuration and the proposed channel combination across all six cross‐dataset combinations. Detailed quantitative results of all 16 channel combinations using all the evaluated models are reported with all the segmentation metrics, including accuracy, precision, and recall, along with results for all six cross‐dataset validation combinations across the three datasets are provided in the Supporting Information (Tables S16, S17, S18, S19, S20, S21, S22, S23, S24, S25, S26, S27, S28, S29, S30, S31, S32, S33, S34, S35, S36, S37, S38, S39, S40, S41, S42, S43, S44, and S45).

## 6. Comparison With the Existing Methods

In order to demonstrate the effectiveness of the proposed segmentation approach, a comparison is conducted with several prominent SOTA models reported in recent literature on lung segmentation across standard benchmark datasets. This task has been widely explored using DL models, including foundational models such as the U‐Net by Ronneberger, which have become a cornerstone of biomedical segmentation. Later studies extended this architecture to improve the boundary accuracy and overall segmentation performance.

Several studies have reported promising outcomes on publicly accessible chest x‐ray datasets, including JSRT, MC, and Shenzhen datasets, either individually or in combination. One notable contribution, a CNN‐based architecture proposed by Sulaiman et al., achieved a dice score of approximately 0.96 and an IoU of 0.93, demonstrating the potential of CNNs for accurate lung segmentation. Similarly, Arvind et al. [36] proposed a lightweight U‐Net architecture for lung segmentation and evaluated it on a combined dataset (JSRT, MC, and SH) and achieved a testing dice and accuracy of 0.9187% and 93.87%, respectively, with reduced architectural complexity through the efficient parameter design. Another recent study by Turq and Kılıçaslan [35] introduced modified U‐Net, V‐Net, and SegNet models to enhance the lung region extraction to detect tuberculosis from CXR images. The models were evaluated on the SH and MC datasets, where the improved U‐Net and V‐Net achieved dice scores of 0.9643 and 0.942 with accuracy values around 0.9824, confirming the effectiveness of encoder–decoder architectures for stable segmentation performance. Another significant transformer‐based study by Khorasani and Mofrad [17] proposed the feature aggregation transformer network (FAT‐Net) architecture, evaluated on the public CXR dataset, and achieved an accuracy of 98.12% and a dice of 96.10%, highlighting the effectiveness of an adaptive feature learning mechanism for accurate and robust lung segmentation.

Overall, the existing studies discussed above indicate that improvements in lung segmentation performance are largely driven by architectural modifications to the U‐Net or by incorporating advanced mechanisms like attention gates, residual blocks, transformer‐based modules, and multiscale feature extraction. Despite these advancements, maintaining consistent performance across heterogeneous datasets remains a persistent challenge, largely because existing studies confine their evaluation to a single dataset or combined datasets without accounting for domain shifts and differences in imaging protocols, leaving cross‐dataset generalization unexplored. To bridge this gap, the proposed approach combines consistent channel combination (*I*_*o*__*c* *n*2_log_pm) using deep attention U‐Net and validates model under both within‐dataset and interdataset evaluation. The proposed approach is evaluated individually on three benchmark datasets, JSRT, MC, and SH achieving dice scores of 0.9814, 0.9782, and 0.9580, along with IoU scores of 0.9638, 0.9577, and 0.9200, respectively, on the separate test sets, confirming competitive performance against existing SOTA methods. The quantitative comparison of the proposed approach with other representative SOTA methods is summarized in Table 8, where the segmentation‐specific metrics are reported.

**Table 8:** Comparison of the proposed approach with the state‐of‐the‐art approaches.

Model/Reference	Author/Year	Dataset	Accuracy	DSC	IoU	Precision	Recall
FAT‐Net [17]	Khorasani and Mofrad (2024)	MC_SH	98.12	96.10	92.62	96.30	96.09
CNN [34]	Sulaiman et al. (2022)	MC_SH	0.97	0.96	0.93	—	—
U‐Net			0.9824	0.9643			
V‐Net	Turk et al. (2025)	MC_SH	0.9824	0.9642	—	—	—
SegNet [35]			0.9834	0.9551			
AMRU++ [16]	Alam et al. (2024)	SH	—	0.9413	0.8916	—	—
Modified U‐Net [36]	Arvind et al. (2023)	JSRT	0.9387	0.9187	—	—	—
		MC					
		SH					
CSR‐Net [37]	Kumar et al. (2023)	JSRT	96.67	94.02	—	—	—
DAA‐UNet [38]	Yadav et al. (2025)	SH	97.15%	93.25%	92.37%	—	—
Swin‐UNet [39]	Yao et al. (2024)	SH	97.80%	95.71%	91.88%	96.81%	94.79%
Deep Attention U‐Net	Proposed method	JSRT	**0.9883**	**0.9815**	**0.9624**	**0.9787**	**0.9823**
	(*I*_*o*__cn_2__log_pm)	MC	**0.9888**	**0.9782**	**0.9577**	**0.9849**	**0.9718**
		SH	**0.9793**	**0.9580**	**0.9200**	**0.9577**	**0.9593**

*Note:* The bolded values indicate the highest segmentation performance among all the compared methods. These values correspond to the best dice and IoU scores achieved by the proposed method across the evaluated datasets, highlighting its superiority over existing approaches.

## 7. Discussion

To examine the impact of edge‐assisted features on lung segmentation, multiple channel combinations were systematically evaluated using all DL architectures considered in this study across all the datasets. Among all tested configurations, a few channel combinations, as mentioned in Tables 3 and 4, demonstrated consistently reliable and stable behavior over the baseline results across all the dataset settings, whether evaluated within or cross dataset validation, confirming that edge‐assisted representations generally provide useful and meaningful guidance to segmentation networks. The findings mentioned in Tables 3 and 4 directly support that multichannel feature refinement provides anatomically meaningful priors that enhance the segmentation performance, thereby addressing RQ1. Another noteworthy finding from the experiments is that the standard U‐Net model showed the highest performance gain when multichannel features were incorporated, as mentioned in Tables 3 and 6. This improvement confirms that the simple encoder–decoder architecture of U‐Net proved especially more responsive to explicit boundary‐enhanced inputs compared with advanced architectures, such as ResU‐Net and attention‐based variants, which already include built‐in feature refinement mechanisms, therefore supporting the RQ2. In contrast, despite having comparatively lower gain from the incorporation of multichannel features, the deep attention U‐Net consistently obtained the highest overall segmentation performance in all datasets. This can be attributed to the model′s inherent ability to utilize the handcrafted‐heuristic features more effectively by its richer architecture and attention mechanism, meaning that the network can focus more precisely on lung boundary information, resulting in excellent performance over all other evaluated models. Beyond the architecture‐specific observations, a particularly interesting effect was observed under cross‐dataset validation, where the baseline channel relying solely on original images only exhibited noticeable performance degradation over all the channel combinations for all the evaluated models, as detailed in the Supporting Information (Tables S16, S17, S18, S19, S20, S21, S22, S23, S24, S25, S26, S27, S28, S29, S30, S31, S32, S33, S34, S35, S36, S37, S38, S39, S40, S41, S42, S43, S44, and S45). The cross‐dataset results presented in Tables 5, 6 and 7 clearly illustrate this improvement, showing that the proposed channel combination consistently outperformed the baseline configuration across all the transfer settings. The gain was measured using the DSC and IoU, as both are broadly accepted as the gold standard for evaluating segmentation quality in medical imaging, collectively providing a strong and positive response to the RQ3 of this study.

Beyond the research questions addressed above, the ablation analysis provides useful insights into the contribution of individual structural features. The findings suggest that canny edges have a great impact on cross‐dataset generalizability. The canny variants capture the fine lung boundary details that enhance localization accuracy. The LoG feature provides a smoother structural representation of the lung region. The spine removal eliminates the strong central edges that would otherwise be incorrectly interpreted as lung boundaries. These observations indicate that all structural features contribute meaningfully to the overall segmentation performance.

In lung segmentation tasks, publicly available datasets are typically provided with the expert‐annotated ground truth masks generated by experienced pulmonologists. It is worth noting that in the present study, segmentation performance is evaluated by comparing the predicted masks against these expert‐annotated reference masks using the standard quantitative metrics. Therefore, the ground truth labels reflect expert clinical knowledge, providing an indirect form of expert validation through the evaluation process.

Overall, all the findings indicate that the proposed edge‐assisted segmentation approach effectively improves the LFS performance.

## 8. Limitations and Future Work

Although the proposed approach demonstrated consistent improvements in LFS performance across multiple chest x‐ray datasets, several limitations remain that open directions for future work. The heuristic feature extraction stage includes some of the scaling constants, which are partially predefined. It may be possible to explore more adaptive parameter estimation strategies based on image histogram characteristics that could further improve cross‐domain performance, and this direction would be worth exploring in future studies. Moreover, as the proposed approach shows promising results across multiple datasets, direct clinical validation by radiologists has not been conducted in this study. However, the present study relies on the expert‐curated ground truth references as an indirect form of validation.

Future research will focus on extending the proposed approach to the volumetric 3D imaging modalities, including CT and MRI scans. Furthermore, recent advancements in explainable AI have gained significant attention in medical imaging to improve the interpretability and transparency of DL models. Future work will incorporate XAI to better understand the segmentation outcomes and aid clinical decision‐making in complex diagnostic situations. Collaboration with expert pulmonologists will additionally validate the segmented lung regions, particularly in cases involving complex pathological conditions, which would provide deeper insights into the practical applicability of the proposed approach.

## 9. Conclusion

This study proposed a two‐stage approach for LFS using chest x‐ray images, wherein the first stage, multiscale heuristic features were extracted by applying the classical image processing techniques, including Canny edge detection and LoG algorithms, which were further refined by morphological operations, to generate structurally enriched representations without requiring any ground truth supervision. These structural features underwent early fusion with the original CXR images to produce multiple multichannel input configurations. In Stage 2, these configurations were passed through a learnable channel mixer before being used to train and evaluate multiple U‐Net–based DL models under both within‐dataset and interdataset validation settings, with a separate held‐out test set to reduce the risk of overfitting. Among all the evaluated configurations, a few channel combinations consistently produced stable segmentation performance across all the dataset settings, evaluated on different models, achieving competitive dice and IoU scores that outperformed existing SOTA methods. More importantly, the approach maintained stable segmentation performance under cross‐dataset validation, where the baseline configuration showed considerable performance decline on completely unseen datasets, highlighting the practical value for real‐world clinical deployment across the varying imaging equipment and acquisition protocols. The findings collectively confirm that the proposed multichannel feature fusion enhances the segmentation performance, boundary delineation, and model robustness across diverse clinical datasets, establishing it as a reliable and effective solution for lung segmentation in chest x‐ray images.

## Funding

No funding was received for this manuscript.

## Ethics Statement

This study used publicly available chest x‐ray datasets and did not involve any interaction with human participants or animals. Therefore, ethical approval was not required.

## Conflicts of Interest

The authors declare no conflicts of interest.

## Supporting information


**Supporting Information** Additional supporting information can be found online in the Supporting Information section. **Supporting Information.** The following Supporting Information accompanies this manuscript. Tables S1–S45: This tables provide detailed quantitative results of all 16 channel combinations evaluated using different U‐Net based models across the JSRT, Montgomery County, and Shenzhen Hospital datasets under both within‐dataset and cross‐dataset validation settings, reported in terms of dice score, IoU, accuracy, precision, and recall.

## Data Availability

The datasets used in this study are publicly available from their respective sources. These include the JSRT dataset (https://www.kaggle.com/datasets/abduzzami/jsrt-247-image-lung-segmentation-mask-dataset), the Montgomery dataset (https://www.kaggle.com/datasets/raddar/tuberculosis-chest-xrays-montgomery), and the Shenzhen dataset (https://www.kaggle.com/datasets/raddar/tuberculosis-chest-xrays-shenzhen).
