# Supplementary material for: Enhancing the Generalizability of Deep Learning–Based Models for Lung Field Segmentation in Chest Radiographs Using Edge‐Assisted Multiscale Feature Fusion
Source: Int J Biomed Imaging. 2026 May 7;2026:6566262. doi: 10.1155/ijbi/6566262 (PMC13150971; doi:10.1155/ijbi/6566262)
Supplement: Supplementary file 1 — Supporting Information Additional supporting information can be found online in the Supporting Information section. Supporting Information. The following Supporting Information accompanies this manuscript. Tables S1–S45: This tables provide detailed quantitative results of all 16 channel combinations evaluated using different U‐Net based models across the JSRT, Montgomery County, and Shenzhen Hospital datasets under both within‐dataset and cross‐dataset validation settings, reported in terms of dice score, IoU, accuracy, precision, and recall. [file IJBI-2026-6566262-s001.docx]

# Supplementary Material

## Title: Enhancing the Generalizability of Deep Learning-Based Models for Lung Field Segmentation in Chest Radiographs using Edge-Assisted Multi-Scale Feature Fusion

The supplementary results are organized into two major sections:

1. Within-Dataset Evaluation
2. Cross-Dataset Validation

### S1. Within-Dataset Segmentation Results

#### S1.1 JSRT Dataset

##### Tables S1–S5 present the segmentation results obtained on the JSRT dataset.

#### Table S1. Within-dataset segmentation performance of U-Net on the JSRT dataset using different structural channel combinations.

| accuracy | precision | recall | dice | IoU | experiment_name |
| --- | --- | --- | --- | --- | --- |
| 0.96233 | 0.92407 | 0.95313 | 0.93792 | 0.88370 | Io__cn1 |
| 0.95250 | 0.96925 | 0.87098 | 0.91709 | 0.84709 | Io__cn1__cn2 |
| 0.96688 | 0.93790 | 0.95318 | 0.94516 | 0.89625 | Io__cn1__cn2__log |
| 0.97466 | 0.95587 | 0.95983 | 0.95766 | 0.91890 | Io__cn1__cn2__log__pm |
| 0.97138 | 0.94069 | 0.96518 | 0.95259 | 0.90968 | Io__cn1__cn2__pm |
| 0.96696 | 0.93695 | 0.95454 | 0.94531 | 0.89663 | Io__cn1__log |
| 0.97093 | 0.93530 | 0.97012 | 0.95222 | 0.90896 | Io__cn1__log__pm |
| 0.97392 | 0.95797 | 0.95498 | 0.95632 | 0.91642 | Io__cn1__pm |
| 0.95975 | 0.95028 | 0.91440 | 0.93170 | 0.87235 | Io__cn2 |
| 0.96751 | 0.94106 | 0.95137 | 0.94594 | 0.89764 | Io__cn2__log |
| 0.97389 | 0.96169 | 0.95080 | 0.95604 | 0.91592 | Io__cn2__log__pm |
| 0.97109 | 0.94150 | 0.96348 | 0.95221 | 0.90891 | Io__cn2__pm |
| 0.94690 | 0.97136 | 0.84930 | 0.90591 | 0.82835 | Io__log |
| 0.96868 | 0.97467 | 0.91971 | 0.94623 | 0.89812 | Io__log__pm |
| 0.97340 | 0.94541 | 0.96709 | 0.95597 | 0.91579 | Io__pm |
| 0.94860 | 0.91794 | 0.91112 | 0.91390 | 0.84209 | Io |

#### Table S2. Within-dataset segmentation performance of ResUNet on the JSRT dataset using different structural channel combinations.

| accuracy | precision | recall | dice | IoU | experiment_name |
| --- | --- | --- | --- | --- | --- |
| 0.98772 | 0.98029 | 0.97869 | 0.97946 | 0.95980 | Io__cn1 |
| 0.98760 | 0.97859 | 0.98000 | 0.97927 | 0.95942 | Io__cn1__cn2 |
| 0.98782 | 0.97866 | 0.98074 | 0.97967 | 0.96020 | Io__cn1__cn2__log |
| 0.98775 | 0.97734 | 0.98172 | 0.97950 | 0.95987 | Io__cn1__cn2__log__pm |
| 0.98759 | 0.97771 | 0.98073 | 0.97919 | 0.95928 | Io__cn1__cn2__pm |
| 0.98747 | 0.97633 | 0.98188 | 0.97907 | 0.95904 | Io__cn1__log |
| 0.98759 | 0.97650 | 0.98214 | 0.97927 | 0.95945 | Io__cn1__log__pm |
| 0.98772 | 0.97866 | 0.98031 | 0.97946 | 0.95979 | Io__cn1__pm |
| 0.98803 | 0.97941 | 0.98060 | 0.97998 | 0.96080 | Io__cn2 |
| 0.98751 | 0.97639 | 0.98208 | 0.97920 | 0.95929 | Io__cn2__log |
| 0.98772 | 0.97719 | 0.98184 | 0.97948 | 0.95984 | Io__cn2__log__pm |
| 0.98748 | 0.97924 | 0.97883 | 0.97900 | 0.95893 | Io__cn2__pm |
| 0.98785 | 0.97931 | 0.98016 | 0.97970 | 0.96026 | Io__log |
| 0.98755 | 0.97703 | 0.98124 | 0.97909 | 0.95911 | Io__log__pm |
| 0.98779 | 0.97743 | 0.98182 | 0.97959 | 0.96005 | Io__pm |
| 0.98785 | 0.97811 | 0.98136 | 0.97970 | 0.96026 | Io |

#### Table S3. Within-dataset segmentation performance of UNet++ on the JSRT dataset using different structural channel combinations.

| accuracy | precision | recall | Dice | IoU | experiment_name |
| --- | --- | --- | --- | --- | --- |
| 0.98802 | 0.98127 | 0.97869 | 0.97996 | 0.96076 | Io__cn1 |
| 0.98814 | 0.97817 | 0.98220 | 0.98015 | 0.96113 | Io__cn1__cn2 |
| 0.98837 | 0.98058 | 0.98063 | 0.98058 | 0.96196 | Io__cn1__cn2__log |
| 0.98791 | 0.97548 | 0.98429 | 0.97983 | 0.96051 | Io__cn1__cn2__log__pm |
| 0.97675 | 0.96469 | 0.95806 | 0.96108 | 0.92536 | Io__cn1__cn2__pm |
| 0.98812 | 0.97761 | 0.98278 | 0.98016 | 0.96115 | Io__cn1__log |
| 0.98764 | 0.97524 | 0.98365 | 0.97940 | 0.95968 | Io__cn1__log__pm |
| 0.98755 | 0.97756 | 0.98081 | 0.97915 | 0.95922 | Io__cn1__pm |
| 0.98815 | 0.97780 | 0.98267 | 0.98100 | 0.96141 | Io__cn2 |
| 0.90345 | 0.76445 | 0.98173 | 0.85883 | 0.75384 | Io__cn2__log |
| 0.98763 | 0.97322 | 0.98575 | 0.97942 | 0.95972 | Io__cn2__log__pm |
| 0.98812 | 0.97685 | 0.98359 | 0.98018 | 0.96119 | Io__cn2__pm |
| 0.98788 | 0.97579 | 0.98401 | 0.97986 | 0.96057 | Io__log |
| 0.98808 | 0.97967 | 0.98054 | 0.98008 | 0.96099 | Io__log__pm |
| 0.98799 | 0.97815 | 0.98165 | 0.97986 | 0.96059 | Io__pm |
| 0.98798 | 0.97717 | 0.98283 | 0.97997 | 0.96078 | Io |

#### Table S4. Within-dataset segmentation performance of Attention Dense U-Net on the JSRT dataset using different structural channel combinations.

| accuracy | Precision | recall | dice | IoU | experiment_name |
| --- | --- | --- | --- | --- | --- |
| 0.98757 | 0.97759 | 0.98106 | 0.97929 | 0.95947 | Io__cn1 |
| 0.98793 | 0.97901 | 0.98067 | 0.97981 | 0.96047 | Io__cn1__cn2 |
| 0.98781 | 0.98063 | 0.97861 | 0.97959 | 0.96004 | Io__cn1__cn2__log |
| 0.98779 | 0.98059 | 0.97853 | 0.97952 | 0.95991 | Io__cn1__cn2__log__pm |
| 0.98769 | 0.97892 | 0.97993 | 0.97939 | 0.95965 | Io__cn1__cn2__pm |
| 0.98745 | 0.97478 | 0.98351 | 0.97910 | 0.95909 | Io__cn1__log |
| 0.98791 | 0.98170 | 0.97790 | 0.97976 | 0.96038 | Io__cn1__log__pm |
| 0.98756 | 0.97597 | 0.98263 | 0.97926 | 0.95941 | Io__cn1__pm |
| 0.98769 | 0.97748 | 0.98155 | 0.97948 | 0.95984 | Io__cn2 |
| 0.98771 | 0.97753 | 0.98159 | 0.97952 | 0.95991 | Io__cn2__log |
| 0.98728 | 0.97614 | 0.98133 | 0.97869 | 0.95833 | Io__cn2__log__pm |
| 0.98769 | 0.97872 | 0.98014 | 0.97940 | 0.95968 | Io__cn2__pm |
| 0.98764 | 0.97661 | 0.98223 | 0.97938 | 0.95964 | Io__log |
| 0.98712 | 0.97472 | 0.98239 | 0.97851 | 0.95797 | Io__log__pm |
| 0.98756 | 0.97665 | 0.98190 | 0.97924 | 0.95937 | Io__pm |
| 0.98783 | 0.97956 | 0.97986 | 0.97968 | 0.96021 | Io |

#### Table S5. Within-dataset segmentation performance of Deep Attention U-Net on the JSRT dataset using different structural channel combinations.

| accuracy | precision | recall | dice | IoU | experiment_name |
| --- | --- | --- | --- | --- | --- |
| 0.98837 | 0.98045 | 0.98078 | 0.98058 | 0.96195 | Io__cn1 |
| 0.98845 | 0.98014 | 0.98135 | 0.98071 | 0.96221 | Io__cn1__cn2 |
| 0.98784 | 0.97527 | 0.98427 | 0.97971 | 0.96028 | Io__cn1__cn2__log |
| 0.98839 | 0.98078 | 0.98042 | 0.98057 | 0.96193 | Io__cn1__cn2__log__pm |
| 0.98820 | 0.97788 | 0.98273 | 0.98026 | 0.96135 | Io__cn1__cn2__pm |
| 0.98805 | 0.97858 | 0.98165 | 0.98007 | 0.96097 | Io__cn1__log |
| 0.98834 | 0.98251 | 0.97856 | 0.98050 | 0.96179 | Io__cn1__log__pm |
| 0.98793 | 0.97667 | 0.98320 | 0.97989 | 0.96062 | Io__cn1__pm |
| 0.98794 | 0.97708 | 0.98282 | 0.97991 | 0.96067 | Io__cn2 |
| 0.98835 | 0.97696 | 0.98435 | 0.98061 | 0.96200 | Io__cn2__log |
| 0.98833 | 0.97874 | 0.98233 | 0.98150 | 0.96249 | Io__cn2__log__pm |
| 0.98838 | 0.97793 | 0.98338 | 0.98062 | 0.96202 | Io__cn2__pm |
| 0.98792 | 0.97632 | 0.98357 | 0.97991 | 0.96064 | Io__log |
| 0.98826 | 0.97749 | 0.98347 | 0.98043 | 0.96167 | Io__log__pm |
| 0.98808 | 0.97723 | 0.98307 | 0.98011 | 0.96105 | Io__pm |
| 0.98825 | 0.98003 | 0.98080 | 0.98039 | 0.96159 | Io |

#### **S1.2 Montgomery County**

Tables S6–S10 report the segmentation results obtained on the Montgomery County dataset.

#### Table S6. Within-dataset segmentation performance of U-Net on the Montgomery dataset using different structural channel combinations.

| accuracy | precision | recall | dice | IoU | experiment_name |
| --- | --- | --- | --- | --- | --- |
| 0.95838 | 0.92050 | 0.91805 | 0.91898 | 0.85284 | Io__cn1 |
| 0.96394 | 0.95608 | 0.90317 | 0.92847 | 0.86778 | Io__cn1__cn2 |
| 0.96482 | 0.91618 | 0.95269 | 0.93386 | 0.87695 | Io__cn1__cn2__log |
| 0.96511 | 0.96878 | 0.89546 | 0.92996 | 0.86985 | Io__cn1__cn2__log__pm |
| 0.96640 | 0.96429 | 0.90518 | 0.93324 | 0.87550 | Io__cn1__cn2__pm |
| 0.95426 | 0.96061 | 0.85985 | 0.90666 | 0.83156 | Io__cn1__log |
| 0.97094 | 0.95356 | 0.93465 | 0.94359 | 0.89373 | Io__cn1__log__pm |
| 0.95669 | 0.99043 | 0.84212 | 0.90929 | 0.83509 | Io__cn1__pm |
| 0.96299 | 0.93722 | 0.91850 | 0.92752 | 0.86702 | Io__cn2 |
| 0.95819 | 0.96918 | 0.86884 | 0.91581 | 0.84570 | Io__cn2__log |
| 0.96695 | 0.94975 | 0.92314 | 0.93567 | 0.87958 | Io__cn2__log__pm |
| 0.96730 | 0.97337 | 0.89955 | 0.93448 | 0.87770 | Io__cn2__pm |
| 0.95934 | 0.93549 | 0.90615 | 0.92015 | 0.85440 | Io__log |
| 0.96846 | 0.95658 | 0.92162 | 0.93823 | 0.88417 | Io__log__pm |
| 0.96556 | 0.97929 | 0.88672 | 0.93001 | 0.87006 | Io__pm |
| 0.95704 | 0.92159 | 0.91075 | 0.91580 | 0.84789 | Io |

#### Table S7. Within-dataset segmentation performance of ResUNet on the Montgomery dataset using different structural channel combinations.

| accuracy | precision | recall | dice | IoU | experiment_name |
| --- | --- | --- | --- | --- | --- |
| 0.98676 | 0.98533 | 0.96349 | 0.97407 | 0.94991 | Io__cn1 |
| 0.98699 | 0.98775 | 0.96177 | 0.97431 | 0.95040 | Io__cn1__cn2 |
| 0.98320 | 0.95837 | 0.97905 | 0.96849 | 0.93937 | Io__cn1__cn2__log |
| 0.98665 | 0.98241 | 0.96639 | 0.97416 | 0.95004 | Io__cn1__cn2__log__pm |
| 0.98721 | 0.98425 | 0.96676 | 0.97528 | 0.95208 | Io__cn1__cn2__pm |
| 0.98649 | 0.98409 | 0.96368 | 0.97355 | 0.94891 | Io__cn1__log |
| 0.98716 | 0.98335 | 0.96746 | 0.97514 | 0.95186 | Io__cn1__log__pm |
| 0.98705 | 0.98303 | 0.96705 | 0.97479 | 0.95122 | Io__cn1__pm |
| 0.98697 | 0.98522 | 0.96461 | 0.97457 | 0.95087 | Io__cn2 |
| 0.98597 | 0.98134 | 0.96491 | 0.97290 | 0.94771 | Io__cn2__log |
| 0.98760 | 0.98713 | 0.96485 | 0.97564 | 0.95281 | Io__cn2__log__pm |
| 0.98731 | 0.98492 | 0.96609 | 0.97519 | 0.95195 | Io__cn2__pm |
| 0.98713 | 0.98703 | 0.96342 | 0.97489 | 0.95140 | Io__log |
| 0.98724 | 0.98458 | 0.96615 | 0.97510 | 0.95182 | Io__log__pm |
| 0.98749 | 0.98328 | 0.96852 | 0.97569 | 0.95288 | Io__pm |
| 0.98604 | 0.98585 | 0.96047 | 0.97276 | 0.94748 | Io |

#### Table S8. Within-dataset segmentation performance of UNet++ on the Montgomery dataset using different structural channel combinations.

| accuracy | precision | recall | dice | iou | experiment_name |
| --- | --- | --- | --- | --- | --- |
| 0.98803 | 0.98592 | 0.96786 | 0.97666 | 0.95474 | Io__cn1 |
| 0.98810 | 0.98750 | 0.96624 | 0.97660 | 0.95463 | Io__cn1__cn2 |
| 0.98706 | 0.97889 | 0.97092 | 0.97463 | 0.95097 | Io__cn1__cn2__log |
| 0.98823 | 0.98765 | 0.96624 | 0.97667 | 0.95481 | Io__cn1__cn2__log__pm |
| 0.98866 | 0.98623 | 0.97005 | 0.97794 | 0.95711 | Io__cn1__cn2__pm |
| 0.98828 | 0.98544 | 0.96864 | 0.97675 | 0.95494 | Io__cn1__log |
| 0.98713 | 0.98623 | 0.96429 | 0.97498 | 0.95149 | Io__cn1__log__pm |
| 0.98759 | 0.98368 | 0.96848 | 0.97581 | 0.95317 | Io__cn1__pm |
| 0.98852 | 0.98399 | 0.97115 | 0.97752 | 0.95614 | Io__cn2 |
| 0.98607 | 0.98688 | 0.95953 | 0.97271 | 0.94754 | Io__cn2__log |
| 0.98741 | 0.98307 | 0.96821 | 0.97534 | 0.95230 | Io__cn2__log__pm |
| 0.98820 | 0.98243 | 0.97176 | 0.97692 | 0.95520 | Io__cn2__pm |
| 0.98449 | 0.98709 | 0.95354 | 0.96981 | 0.94239 | Io__log |
| 0.98673 | 0.99002 | 0.95841 | 0.97373 | 0.94933 | Io__log__pm |
| 0.98725 | 0.98131 | 0.96937 | 0.97509 | 0.95179 | Io__pm |
| 0.98726 | 0.98138 | 0.96937 | 0.97517 | 0.95201 | Io |

#### Table S9. Within-dataset segmentation performance of Attention Dense U-Net on the Montgomery dataset using different structural channel combinations.

| accuracy | precision | recall | dice | IoU | experiment_name |
| --- | --- | --- | --- | --- | --- |
| 0.98623 | 0.98027 | 0.96700 | 0.97341 | 0.94884 | Io__cn1 |
| 0.98577 | 0.97192 | 0.97302 | 0.97229 | 0.94653 | Io__cn1__cn2 |
| 0.98668 | 0.98349 | 0.96570 | 0.97435 | 0.95056 | Io__cn1__cn2__log |
| 0.98723 | 0.98462 | 0.96614 | 0.97510 | 0.95183 | Io__cn1__cn2__log__pm |
| 0.98755 | 0.98079 | 0.97121 | 0.97585 | 0.95313 | Io__cn1__cn2__pm |
| 0.98701 | 0.97824 | 0.97191 | 0.97492 | 0.95149 | Io__cn1__log |
| 0.98715 | 0.97628 | 0.97451 | 0.97530 | 0.95219 | Io__cn1__log__pm |
| 0.98619 | 0.97161 | 0.97624 | 0.97387 | 0.94942 | Io__cn1__pm |
| 0.98543 | 0.98251 | 0.96207 | 0.97196 | 0.94611 | Io__cn2 |
| 0.98760 | 0.98211 | 0.97029 | 0.97605 | 0.95359 | Io__cn2__log |
| 0.98699 | 0.98015 | 0.97001 | 0.97493 | 0.95150 | Io__cn2__log__pm |
| 0.98812 | 0.98032 | 0.97370 | 0.97688 | 0.95508 | Io__cn2__pm |
| 0.98290 | 0.97021 | 0.96460 | 0.96719 | 0.93738 | Io__log |
| 0.98765 | 0.97759 | 0.97509 | 0.97623 | 0.95384 | Io__log__pm |
| 0.98763 | 0.98180 | 0.97061 | 0.97605 | 0.95359 | Io__pm |
| 0.98670 | 0.97857 | 0.97027 | 0.97425 | 0.95025 | Io |

#### Table S10. Within-dataset segmentation performance of Deep Attention U-Net on the Montgomery dataset using different structural channel combinations.

| accuracy | precision | recall | dice | IoU | experiment_name |
| --- | --- | --- | --- | --- | --- |
| 0.98626 | 0.98234 | 0.96551 | 0.97357 | 0.94925 | Io__cn1 |
| 0.98823 | 0.98528 | 0.96894 | 0.97687 | 0.95519 | Io__cn1__cn2 |
| 0.98687 | 0.98105 | 0.96903 | 0.97481 | 0.95142 | Io__cn1__cn2__log |
| 0.98773 | 0.98193 | 0.97059 | 0.97601 | 0.95355 | Io__cn1__cn2__log__pm |
| 0.98847 | 0.98290 | 0.97274 | 0.97771 | 0.95664 | Io__cn1__cn2__pm |
| 0.98760 | 0.98343 | 0.96864 | 0.97579 | 0.95318 | Io__cn1__log |
| 0.98694 | 0.97934 | 0.97046 | 0.97475 | 0.95113 | Io__cn1__log__pm |
| 0.98684 | 0.98551 | 0.96367 | 0.97423 | 0.95027 | Io__cn1__pm |
| 0.98616 | 0.97993 | 0.96711 | 0.97319 | 0.94837 | Io__cn2 |
| 0.98614 | 0.98311 | 0.96423 | 0.97330 | 0.94872 | Io__cn2__log |
| 0.98884 | 0.98498 | 0.97188 | 0.97825 | 0.95775 | Io__cn2__log__pm |
| 0.98825 | 0.98373 | 0.97094 | 0.97713 | 0.95562 | Io__cn2__pm |
| 0.98701 | 0.98304 | 0.96658 | 0.97458 | 0.95085 | Io__log |
| 0.98728 | 0.98189 | 0.96931 | 0.97538 | 0.95233 | Io__log__pm |
| 0.98715 | 0.98471 | 0.96553 | 0.97485 | 0.95146 | Io__pm |
| 0.98825 | 0.98442 | 0.97025 | 0.97714 | 0.95562 | Io |

#### **S1.3 Shenzhen Dataset**

Tables S11–S15 present the segmentation results obtained on the Shenzhen dataset.

#### Table S11. Within-dataset segmentation performance of U-Net on the Shenzhen dataset using different structural channel combinations.

| accuracy | precision | recall | dice | iou | experiment_name |
| --- | --- | --- | --- | --- | --- |
| 0.97231 | 0.95945 | 0.92832 | 0.94264 | 0.89264 | Io__cn1 |
| 0.97016 | 0.93023 | 0.95183 | 0.94007 | 0.88768 | Io__cn1__cn2 |
| 0.97070 | 0.96348 | 0.91717 | 0.93875 | 0.88581 | Io__cn1__cn2__log |
| 0.97408 | 0.94999 | 0.94583 | 0.94716 | 0.90038 | Io__cn1__cn2__log__pm |
| 0.97305 | 0.96140 | 0.92920 | 0.94429 | 0.89543 | Io__cn1__cn2__pm |
| 0.97158 | 0.95934 | 0.92545 | 0.94118 | 0.89000 | Io__cn1__log |
| 0.97275 | 0.95867 | 0.93095 | 0.94377 | 0.89453 | Io__cn1__log__pm |
| 0.97238 | 0.95846 | 0.92959 | 0.94296 | 0.89300 | Io__cn1__pm |
| 0.97193 | 0.95218 | 0.93456 | 0.94241 | 0.89197 | Io__cn2 |
| 0.97210 | 0.95267 | 0.93475 | 0.94274 | 0.89254 | Io__cn2__log |
| 0.97316 | 0.95325 | 0.93855 | 0.94503 | 0.89675 | Io__cn2__log__pm |
| 0.97404 | 0.96111 | 0.93372 | 0.94644 | 0.89929 | Io__cn2__pm |
| 0.96696 | 0.97205 | 0.89324 | 0.92978 | 0.87039 | Io__log |
| 0.97110 | 0.92772 | 0.95902 | 0.94231 | 0.89169 | Io__log__pm |
| 0.97181 | 0.96364 | 0.92175 | 0.94127 | 0.89041 | Io__pm |
| 0.96436 | 0.97225 | 0.88235 | 0.92367 | 0.86008 | Io |

#### Table S12. Within-dataset segmentation performance of ResUNet on the Shenzhen dataset using different structural channel combinations.

| accuracy | precision | recall | dice | iou | experiment_name |
| --- | --- | --- | --- | --- | --- |
| 0.97972 | 0.97057 | 0.94729 | 0.95830 | 0.92054 | Io__cn1 |
| 0.97979 | 0.96735 | 0.95091 | 0.95856 | 0.92103 | Io__cn1__cn2 |
| 0.97977 | 0.96574 | 0.95245 | 0.95855 | 0.92099 | Io__cn1__cn2__log |
| 0.97971 | 0.96939 | 0.94856 | 0.95837 | 0.92064 | Io__cn1__cn2__log__pm |
| 0.97944 | 0.96710 | 0.94971 | 0.95783 | 0.91962 | Io__cn1__cn2__pm |
| 0.97972 | 0.97039 | 0.94746 | 0.95830 | 0.92053 | Io__cn1__log |
| 0.97969 | 0.97005 | 0.94772 | 0.95824 | 0.92044 | Io__cn1__log__pm |
| 0.97931 | 0.96787 | 0.94844 | 0.95753 | 0.91908 | Io__cn1__pm |
| 0.97947 | 0.96748 | 0.94947 | 0.95792 | 0.91986 | Io__cn2 |
| 0.97962 | 0.97046 | 0.94696 | 0.95804 | 0.92008 | Io__cn2__log |
| 0.97985 | 0.96962 | 0.94878 | 0.95859 | 0.92104 | Io__cn2__log__pm |
| 0.97981 | 0.96809 | 0.95015 | 0.95852 | 0.92090 | Io__cn2__pm |
| 0.97961 | 0.96720 | 0.95026 | 0.95818 | 0.92034 | Io__log |
| 0.97971 | 0.96533 | 0.95269 | 0.95847 | 0.92082 | Io__log__pm |
| 0.97970 | 0.97087 | 0.94690 | 0.95820 | 0.92038 | Io__pm |
| 0.97970 | 0.97021 | 0.94752 | 0.95824 | 0.92044 | Io |

#### Table S13. Within-dataset segmentation performance of UNet++ on the Shenzhen dataset using different structural channel combinations.

| accuracy | precision | recall | dice | iou | experiment_name |
| --- | --- | --- | --- | --- | --- |
| 0.97972 | 0.96946 | 0.94851 | 0.95840 | 0.92071 | Io__cn1 |
| 0.97931 | 0.96262 | 0.95390 | 0.95775 | 0.91945 | Io__cn1__cn2 |
| 0.97927 | 0.96598 | 0.95016 | 0.95751 | 0.91907 | Io__cn1__cn2__log |
| 0.97958 | 0.96549 | 0.95197 | 0.95818 | 0.92026 | Io__cn1__cn2__log__pm |
| 0.97939 | 0.97058 | 0.94583 | 0.95754 | 0.91914 | Io__cn1__cn2__pm |
| 0.97957 | 0.96933 | 0.94797 | 0.95801 | 0.92000 | Io__cn1__log |
| 0.97974 | 0.96957 | 0.94834 | 0.95833 | 0.92056 | Io__cn1__log__pm |
| 0.97982 | 0.97271 | 0.94548 | 0.95837 | 0.92068 | Io__cn1__pm |
| 0.97982 | 0.96682 | 0.95156 | 0.95864 | 0.92114 | Io__cn2 |
| 0.97946 | 0.97230 | 0.94441 | 0.95764 | 0.91933 | Io__cn2__log |
| 0.97941 | 0.97260 | 0.94394 | 0.95752 | 0.91912 | Io__cn2__log__pm |
| 0.97914 | 0.96115 | 0.95482 | 0.95745 | 0.91888 | Io__cn2__pm |
| 0.97928 | 0.96695 | 0.94914 | 0.95747 | 0.91900 | Io__log |
| 0.97902 | 0.96918 | 0.94584 | 0.95687 | 0.91789 | Io__log__pm |
| 0.97923 | 0.96817 | 0.94785 | 0.95740 | 0.91884 | Io__pm |
| 0.97953 | 0.96322 | 0.95399 | 0.95813 | 0.92018 | Io |

#### Table S14. Within-dataset segmentation performance of Attention Dense U-Net on the Shenzhen dataset using different structural channel combinations.

| accuracy | precision | recall | dice | IoU | experiment_name |
| --- | --- | --- | --- | --- | --- |
| 0.97989 | 0.97090 | 0.94759 | 0.95860 | 0.92107 | Io__cn1 |
| 0.97877 | 0.95874 | 0.95568 | 0.95674 | 0.91757 | Io__cn1__cn2 |
| 0.97875 | 0.96439 | 0.94987 | 0.95659 | 0.91734 | Io__cn1__cn2__log |
| 0.97918 | 0.96965 | 0.94591 | 0.95713 | 0.91838 | Io__cn1__cn2__log__pm |
| 0.97925 | 0.97220 | 0.94364 | 0.95720 | 0.91853 | Io__cn1__cn2__pm |
| 0.97929 | 0.96477 | 0.95153 | 0.95761 | 0.91919 | Io__cn1__log |
| 0.97921 | 0.96784 | 0.94811 | 0.95735 | 0.91876 | Io__cn1__log__pm |
| 0.97925 | 0.96492 | 0.95131 | 0.95761 | 0.91922 | Io__cn1__pm |
| 0.97918 | 0.96555 | 0.95031 | 0.95735 | 0.91875 | Io__cn2 |
| 0.97909 | 0.96912 | 0.94610 | 0.95695 | 0.91807 | Io__cn2__log |
| 0.97963 | 0.96966 | 0.94781 | 0.95811 | 0.92018 | Io__cn2__log__pm |
| 0.97850 | 0.95908 | 0.95414 | 0.95614 | 0.91651 | Io__cn2__pm |
| 0.97958 | 0.96794 | 0.94943 | 0.95809 | 0.92012 | Io__log |
| 0.97934 | 0.97105 | 0.94514 | 0.95743 | 0.91890 | Io__log__pm |
| 0.97960 | 0.96606 | 0.95149 | 0.95821 | 0.92034 | Io__pm |
| 0.97929 | 0.96868 | 0.94744 | 0.95745 | 0.91900 | Io |

Table S15. Within-dataset segmentation performance of Deep Attention U-Net on the Shenzhen dataset using different structural channel combinations.

| accuracy | precision | recall | dice | IoU | experiment_name |
| --- | --- | --- | --- | --- | --- |
| 0.97935 | 0.96314 | 0.95345 | 0.95780 | 0.91958 | Io__cn1 |
| 0.97965 | 0.96650 | 0.95123 | 0.95833 | 0.92058 | Io__cn1__cn2 |
| 0.97949 | 0.95957 | 0.95773 | 0.95811 | 0.92013 | Io__cn1__cn2__log |
| 0.97966 | 0.97209 | 0.94548 | 0.95807 | 0.92012 | Io__cn1__cn2__log__pm |
| 0.97950 | 0.97519 | 0.94176 | 0.95771 | 0.91943 | Io__cn1__cn2__pm |
| 0.97963 | 0.96643 | 0.95133 | 0.95831 | 0.92055 | Io__cn1__log |
| 0.97964 | 0.96775 | 0.94977 | 0.95809 | 0.92004 | Io__cn1__log__pm |
| 0.98021 | 0.96872 | 0.95105 | 0.95931 | 0.92245 | Io__cn1__pm |
| 0.97946 | 0.97054 | 0.94619 | 0.95767 | 0.91940 | Io__cn2 |
| 0.97968 | 0.96551 | 0.95235 | 0.95841 | 0.92071 | Io__cn2__log |
| 0.97938 | 0.95771 | 0.95937 | 0.95814 | 0.92031 | Io__cn2__log__pm |
| 0.97979 | 0.96638 | 0.95197 | 0.95863 | 0.92110 | Io__cn2__pm |
| 0.97989 | 0.97117 | 0.94735 | 0.95861 | 0.92113 | Io__log |
| 0.97844 | 0.96566 | 0.94718 | 0.95581 | 0.91597 | Io__log__pm |
| 0.97950 | 0.96462 | 0.95250 | 0.95800 | 0.91999 | Io__pm |
| 0.97924 | 0.96629 | 0.94976 | 0.95747 | 0.91901 | Io |

### **S2. Cross-Dataset Validation Results**

A total of six cross-dataset transfer scenarios were considered:

JSRT → MC

JSRT → SH

MC → JSRT

MC → SH

SH → JSRT

SH → MC

#### **S2.1 JSRT → MC**

#### Table S16. Cross-dataset segmentation performance when training on JSRT and testing on Montgomery using U-Net.

| accuracy | precision | recall | dice | iou | Experiment |
| --- | --- | --- | --- | --- | --- |
| 0.91674 | 0.78314 | 0.92638 | 0.84203 | 0.73509 | Io |
| 0.92241 | 0.81433 | 0.89680 | 0.83627 | 0.73161 | Io__cn1 |
| 0.93081 | 0.81564 | 0.93756 | 0.86379 | 0.76709 | Io__cn2 |
| 0.94284 | 0.85639 | 0.92914 | 0.87886 | 0.79069 | Io__cn1__cn2 |
| 0.93321 | 0.81225 | 0.95615 | 0.87648 | 0.78699 | Io__log |
| 0.94224 | 0.85160 | 0.93365 | 0.88078 | 0.79475 | Io__cn1__log |
| 0.93881 | 0.82551 | 0.96036 | 0.88527 | 0.80041 | Io__cn2__log |
| 0.93336 | 0.80794 | 0.96517 | 0.87920 | 0.78978 | Io__cn1__cn2__log |
| 0.96884 | 0.94635 | 0.92909 | 0.91010 | 0.85477 | Io__pm |
| 0.96887 | 0.93357 | 0.94369 | 0.91842 | 0.86528 | Io__cn1__pm |
| 0.96354 | 0.90615 | 0.95425 | 0.91724 | 0.86057 | Io__cn2__pm |
| 0.96825 | 0.96358 | 0.90843 | 0.90327 | 0.83944 | Io__cn1__cn2__pm |
| 0.96654 | 0.92864 | 0.93952 | 0.90874 | 0.85123 | Io__log__pm |
| 0.96670 | 0.93090 | 0.93753 | 0.91302 | 0.85590 | Io__cn1__log__pm |
| 0.94758 | 0.85051 | 0.96105 | 0.89697 | 0.82142 | Io__cn2__log__pm |
| 0.96630 | 0.92407 | 0.94393 | 0.91702 | 0.85929 | Io__cn1__cn2__log__pm |

#### Table S17. Cross-dataset segmentation performance when training on JSRT and testing on Montgomery using ResUNet.

| accuracy | precision | recall | dice | IoU | Experiment |
| --- | --- | --- | --- | --- | --- |
| 0.97820 | 0.95384 | 0.96002 | 0.95070 | 0.90915 | Io |
| 0.97734 | 0.94918 | 0.96163 | 0.94738 | 0.90389 | Io__cn1 |
| 0.97931 | 0.95932 | 0.95861 | 0.94951 | 0.90741 | Io__cn2 |
| 0.98010 | 0.96166 | 0.95935 | 0.95381 | 0.91382 | Io__cn1__cn2 |
| 0.97450 | 0.94258 | 0.95719 | 0.93551 | 0.88705 | Io__log |
| 0.97783 | 0.95551 | 0.95661 | 0.94862 | 0.90582 | Io__cn1__log |
| 0.97890 | 0.95868 | 0.95762 | 0.94962 | 0.90757 | Io__cn2__log |
| 0.97284 | 0.93584 | 0.95799 | 0.93442 | 0.88552 | Io__cn1__cn2__log |
| 0.98022 | 0.96423 | 0.95705 | 0.95322 | 0.91257 | Io__pm |
| 0.97881 | 0.95764 | 0.95837 | 0.95098 | 0.90931 | Io__cn1__pm |
| 0.97902 | 0.95712 | 0.95982 | 0.95171 | 0.91071 | Io__cn2__pm |
| 0.97886 | 0.95674 | 0.95954 | 0.95113 | 0.90959 | Io__cn1__cn2__pm |
| 0.97569 | 0.94555 | 0.95882 | 0.93999 | 0.89431 | Io__log__pm |
| 0.98008 | 0.96037 | 0.96063 | 0.95287 | 0.91258 | Io__cn1__log__pm |
| 0.98035 | 0.96688 | 0.95480 | 0.94792 | 0.90406 | Io__cn2__log__pm |
| 0.98043 | 0.96421 | 0.95794 | 0.95251 | 0.91210 | Io__cn1__cn2__log__pm |

#### Table S18. Cross-dataset segmentation performance when training on JSRT and testing on Montgomery using U-Net++.

| accuracy | precision | recall | dice | iou | Experiment |
| --- | --- | --- | --- | --- | --- |
| 0.97064 | 0.92781 | 0.95813 | 0.93122 | 0.88258 | Io |
| 0.97792 | 0.95557 | 0.95693 | 0.94734 | 0.90331 | Io__cn1 |
| 0.98094 | 0.96770 | 0.95632 | 0.95343 | 0.91329 | Io__cn2 |
| 0.97406 | 0.94068 | 0.95749 | 0.93832 | 0.89155 | Io__cn1__cn2 |
| 0.97904 | 0.95720 | 0.95978 | 0.95073 | 0.90894 | Io__log |
| 0.97952 | 0.95588 | 0.96326 | 0.95000 | 0.90798 | Io__cn1__log |
| 0.98001 | 0.96776 | 0.95247 | 0.94955 | 0.90775 | Io__cn2__log |
| 0.98131 | 0.96708 | 0.95850 | 0.95586 | 0.91720 | Io__cn1__cn2__log |
| 0.97974 | 0.96270 | 0.95673 | 0.95167 | 0.91031 | Io__pm |
| 0.97899 | 0.95675 | 0.96010 | 0.94775 | 0.90416 | Io__cn1__pm |
| 0.97730 | 0.95360 | 0.95651 | 0.94316 | 0.89776 | Io__cn2__pm |
| 0.97935 | 0.96731 | 0.95021 | 0.94644 | 0.90361 | Io__cn1__cn2__pm |
| 0.98004 | 0.96463 | 0.95590 | 0.95070 | 0.90939 | Io__log__pm |
| 0.98128 | 0.97078 | 0.95448 | 0.95022 | 0.90782 | Io__cn1__log__pm |
| 0.97998 | 0.95705 | 0.96386 | 0.95404 | 0.91561 | Io__cn2__log__pm |
| 0.97937 | 0.96221 | 0.95573 | 0.94827 | 0.90594 | Io__cn1__cn2__log__pm |

#### Table S19. Cross-dataset segmentation performance when training on JSRT and testing on Montgomery using Attention Dense U-Net.

| accuracy | precision | recall | dice | IoU | Experiment |
| --- | --- | --- | --- | --- | --- |
| 0.97371 | 0.94041 | 0.95636 | 0.93548 | 0.88624 | Io |
| 0.97715 | 0.95473 | 0.95465 | 0.94342 | 0.89869 | Io__cn1 |
| 0.97854 | 0.94948 | 0.96633 | 0.95478 | 0.91635 | Io__cn2 |
| 0.97729 | 0.94883 | 0.96183 | 0.94782 | 0.90478 | Io__cn1__cn2 |
| 0.96756 | 0.91312 | 0.96298 | 0.92344 | 0.87061 | Io__log |
| 0.97815 | 0.95319 | 0.96051 | 0.95031 | 0.90769 | Io__cn1__log |
| 0.97379 | 0.93640 | 0.96135 | 0.94070 | 0.89324 | Io__cn2__log |
| 0.97794 | 0.95008 | 0.96314 | 0.95135 | 0.90954 | Io__cn1__cn2__log |
| 0.97820 | 0.95191 | 0.96216 | 0.94710 | 0.90447 | Io__pm |
| 0.97731 | 0.95808 | 0.95164 | 0.94173 | 0.89512 | Io__cn1__pm |
| 0.97771 | 0.95223 | 0.95977 | 0.94873 | 0.90525 | Io__cn2__pm |
| 0.97991 | 0.95858 | 0.96190 | 0.95376 | 0.91390 | Io__cn1__cn2__pm |
| 0.97542 | 0.94367 | 0.95981 | 0.93950 | 0.89277 | Io__log__pm |
| 0.97685 | 0.95134 | 0.95716 | 0.94276 | 0.89673 | Io__cn1__log__pm |
| 0.97070 | 0.92766 | 0.95857 | 0.92826 | 0.87649 | Io__cn2__log__pm |
| 0.98058 | 0.96460 | 0.95814 | 0.95459 | 0.91534 | Io__cn1__cn2__log__pm |

#### Table S20. Cross-dataset segmentation performance when training on JSRT and testing on Montgomery using Deep Attention U-Net.

| Accuracy | precision | recall | dice | IoU | Experiment |
| --- | --- | --- | --- | --- | --- |
| 0.97270 | 0.93673 | 0.95635 | 0.93318 | 0.88491 | Io |
| 0.97939 | 0.96643 | 0.95133 | 0.94706 | 0.90317 | Io__cn1 |
| 0.97652 | 0.95970 | 0.94664 | 0.94321 | 0.90174 | Io__cn2 |
| 0.98163 | 0.96469 | 0.96238 | 0.95735 | 0.92015 | Io__cn1__cn2 |
| 0.98179 | 0.96173 | 0.96622 | 0.95797 | 0.92091 | Io__log |
| 0.97832 | 0.95834 | 0.95556 | 0.94862 | 0.90607 | Io__cn1__log |
| 0.97849 | 0.95676 | 0.95800 | 0.95076 | 0.90941 | Io__cn2__log |
| 0.97643 | 0.95804 | 0.94805 | 0.94238 | 0.89642 | Io__cn1__cn2__log |
| 0.97992 | 0.96296 | 0.95721 | 0.95175 | 0.91027 | Io__pm |
| 0.97931 | 0.96315 | 0.95447 | 0.94917 | 0.90703 | Io__cn1__pm |
| 0.97448 | 0.94663 | 0.95252 | 0.93537 | 0.88451 | Io__cn2__pm |
| 0.97886 | 0.96451 | 0.95116 | 0.94719 | 0.90355 | Io__cn1__cn2__pm |
| 0.97876 | 0.95520 | 0.96085 | 0.95198 | 0.91086 | Io__log__pm |
| 0.97965 | 0.96170 | 0.95743 | 0.94960 | 0.90732 | Io__cn1__log__pm |
| 0.97686 | 0.94972 | 0.95899 | 0.94623 | 0.90211 | Io__cn2__log__pm |
| 0.97913 | 0.96147 | 0.95555 | 0.95049 | 0.90937 | Io__cn1__cn2__log__pm |

#### **S2.2 MC → JSRT**

#### Table S21. Cross-dataset segmentation performance when training on Montgomery and testing on JSRT using U-Net.

| accuracy | precision | Recall | dice | IoU | Experiment |
| --- | --- | --- | --- | --- | --- |
| 0.85821 | 0.82870 | 0.64159 | 0.70278 | 0.54934 | Io |
| 0.87936 | 0.83382 | 0.72711 | 0.77586 | 0.63889 | Io__cn1 |
| 0.92206 | 0.87981 | 0.84559 | 0.86507 | 0.76379 | Io__cn2 |
| 0.92006 | 0.90038 | 0.81310 | 0.85176 | 0.74415 | Io__cn1__cn2 |
| 0.91187 | 0.86748 | 0.82008 | 0.84066 | 0.72807 | Io__log |
| 0.91570 | 0.88170 | 0.81778 | 0.84786 | 0.73859 | Io__cn1__log |
| 0.93503 | 0.87490 | 0.90430 | 0.89507 | 0.81111 | Io__cn2__log |
| 0.92997 | 0.91518 | 0.83486 | 0.87218 | 0.77482 | Io__cn1__cn2__log |
| 0.95455 | 0.91914 | 0.92389 | 0.92132 | 0.85513 | Io__pm |
| 0.95895 | 0.90234 | 0.96197 | 0.93455 | 0.87813 | Io__cn1__pm |
| 0.95084 | 0.88300 | 0.95648 | 0.92560 | 0.86221 | Io__cn2__pm |
| 0.94925 | 0.90826 | 0.91684 | 0.91622 | 0.84646 | Io__cn1__cn2__pm |
| 0.95356 | 0.87799 | 0.97461 | 0.93066 | 0.87115 | Io__log__pm |
| 0.94871 | 0.92495 | 0.89500 | 0.90887 | 0.83440 | Io__cn1__log__pm |
| 0.95289 | 0.87661 | 0.97394 | 0.93162 | 0.87273 | Io__cn2__log__pm |
| 0.95363 | 0.91121 | 0.93004 | 0.92380 | 0.85926 | Io__cn1__cn2__log__pm |

#### Table S22. Cross-dataset segmentation performance when training on Montgomery and testing on JSRT using ResUNet.

| accuracy | precision | recall | dice | IoU | Experiment |
| --- | --- | --- | --- | --- | --- |
| 0.96961 | 0.92415 | 0.97477 | 0.95992 | 0.92326 | Io |
| 0.97147 | 0.92257 | 0.98375 | 0.96259 | 0.92814 | Io__cn1 |
| 0.96859 | 0.91875 | 0.97769 | 0.95938 | 0.92221 | Io__cn2 |
| 0.96540 | 0.90663 | 0.98125 | 0.95682 | 0.91747 | Io__cn1__cn2 |
| 0.96940 | 0.91805 | 0.98165 | 0.96029 | 0.92392 | Io__log |
| 0.97002 | 0.92176 | 0.97931 | 0.96101 | 0.92530 | Io__cn1__log |
| 0.96997 | 0.91489 | 0.98789 | 0.96302 | 0.92891 | Io__cn2__log |
| 0.97144 | 0.91900 | 0.98819 | 0.96481 | 0.93225 | Io__cn1__cn2__log |
| 0.96968 | 0.91304 | 0.98922 | 0.96276 | 0.92855 | Io__pm |
| 0.97001 | 0.91855 | 0.98334 | 0.96098 | 0.92526 | Io__cn1__pm |
| 0.97005 | 0.91950 | 0.98226 | 0.96188 | 0.92688 | Io__cn2__pm |
| 0.97045 | 0.91694 | 0.98707 | 0.96286 | 0.92872 | Io__cn1__cn2__pm |
| 0.97300 | 0.92587 | 0.98540 | 0.96523 | 0.93306 | Io__log__pm |
| 0.97089 | 0.91742 | 0.98813 | 0.96334 | 0.92963 | Io__cn1__log__pm |
| 0.97118 | 0.91842 | 0.98796 | 0.96349 | 0.92992 | Io__cn2__log__pm |
| 0.97088 | 0.91781 | 0.98760 | 0.96377 | 0.93033 | Io__cn1__cn2__log__pm |

#### Table S23. Cross-dataset segmentation performance when training on Montgomery and testing on JSRT using U-Net++.

| accuracy | Precision | recall | dice | IoU | Experiment |
| --- | --- | --- | --- | --- | --- |
| 0.96638 | 0.92005 | 0.96764 | 0.95520 | 0.91477 | Io |
| 0.96973 | 0.92522 | 0.97389 | 0.96005 | 0.92365 | Io__cn1 |
| 0.97143 | 0.92312 | 0.98293 | 0.96370 | 0.93026 | Io__cn2 |
| 0.97193 | 0.92340 | 0.98446 | 0.96443 | 0.93153 | Io__cn1__cn2 |
| 0.96470 | 0.92023 | 0.96107 | 0.95051 | 0.90680 | Io__log |
| 0.96830 | 0.90722 | 0.99162 | 0.96192 | 0.92691 | Io__cn1__log |
| 0.96874 | 0.91427 | 0.98399 | 0.96132 | 0.92578 | Io__cn2__log |
| 0.96785 | 0.91790 | 0.97594 | 0.95789 | 0.91956 | Io__cn1__cn2__log |
| 0.96772 | 0.90553 | 0.99168 | 0.96139 | 0.92596 | Io__pm |
| 0.96908 | 0.91177 | 0.98858 | 0.96251 | 0.92798 | Io__cn1__pm |
| 0.96889 | 0.91311 | 0.98609 | 0.96098 | 0.92521 | Io__cn2__pm |
| 0.97258 | 0.92835 | 0.98073 | 0.96354 | 0.92994 | Io__cn1__cn2__pm |
| 0.97154 | 0.92192 | 0.98486 | 0.96392 | 0.93065 | Io__log__pm |
| 0.97276 | 0.92126 | 0.99030 | 0.96633 | 0.93514 | Io__cn1__log__pm |
| 0.97081 | 0.91644 | 0.98911 | 0.96473 | 0.93217 | Io__cn2__log__pm |
| 0.96905 | 0.90948 | 0.99151 | 0.96299 | 0.92894 | Io__cn1__cn2__log__pm |

#### Table S24. Cross-dataset segmentation performance when training on Montgomery and testing on JSRT using Attention Dense U-Net.

| Accuracy | Precision | recall | dice | IoU | Experiment |
| --- | --- | --- | --- | --- | --- |
| 0.96622 | 0.91623 | 0.97186 | 0.95705 | 0.91800 | Io |
| 0.96951 | 0.92692 | 0.97096 | 0.95895 | 0.92152 | Io__cn1 |
| 0.96638 | 0.92014 | 0.96754 | 0.95588 | 0.91602 | Io__cn2 |
| 0.96547 | 0.90717 | 0.98080 | 0.95674 | 0.91755 | Io__cn1__cn2 |
| 0.96616 | 0.91154 | 0.97767 | 0.95732 | 0.91856 | Io__log |
| 0.96465 | 0.91876 | 0.96270 | 0.95240 | 0.90957 | Io__cn1__log |
| 0.96872 | 0.91271 | 0.98596 | 0.96131 | 0.92574 | Io__cn2__log |
| 0.96874 | 0.91220 | 0.98670 | 0.96107 | 0.92542 | Io__cn1__cn2__log |
| 0.96632 | 0.90193 | 0.99113 | 0.95867 | 0.92105 | Io__pm |
| 0.97079 | 0.91487 | 0.99107 | 0.96449 | 0.93166 | Io__cn1__pm |
| 0.96929 | 0.91258 | 0.98832 | 0.96273 | 0.92840 | Io__cn2__pm |
| 0.96979 | 0.91356 | 0.98897 | 0.96361 | 0.93004 | Io__cn1__cn2__pm |
| 0.97061 | 0.91658 | 0.98814 | 0.96346 | 0.92985 | Io__log__pm |
| 0.96684 | 0.90832 | 0.98453 | 0.95919 | 0.92194 | Io__cn1__log__pm |
| 0.96999 | 0.91377 | 0.98945 | 0.96325 | 0.92938 | Io__cn2__log__pm |
| 0.96820 | 0.90682 | 0.99179 | 0.96258 | 0.92818 | Io__cn1__cn2__log__pm |

#### Table S25. Cross-dataset segmentation performance when training on Montgomery and testing on JSRT using Deep Attention U-Net

| Accuracy | precision | recall | dice | IoU | Experiment |
| --- | --- | --- | --- | --- | --- |
| 0.96998 | 0.91772 | 0.98429 | 0.96219 | 0.92750 | Io |
| 0.96801 | 0.90810 | 0.98934 | 0.96115 | 0.92546 | Io__cn1 |
| 0.96802 | 0.90794 | 0.98960 | 0.96167 | 0.92643 | Io__cn2 |
| 0.96909 | 0.91157 | 0.98888 | 0.96206 | 0.92726 | Io__cn1__cn2 |
| 0.96751 | 0.91519 | 0.97813 | 0.95839 | 0.92050 | Io__log |
| 0.96587 | 0.91499 | 0.97213 | 0.95416 | 0.91283 | Io__cn1__log |
| 0.97016 | 0.92067 | 0.98121 | 0.96319 | 0.92925 | Io__cn2__log |
| 0.96932 | 0.91870 | 0.98053 | 0.96095 | 0.92515 | Io__cn1__cn2__log |
| 0.97301 | 0.92551 | 0.98589 | 0.96556 | 0.93368 | Io__pm |
| 0.96887 | 0.90662 | 0.99462 | 0.96341 | 0.92965 | Io__cn1__pm |
| 0.96842 | 0.91078 | 0.98735 | 0.95951 | 0.92252 | Io__cn2__pm |
| 0.96804 | 0.90629 | 0.99190 | 0.96260 | 0.92814 | Io__cn1__cn2__pm |
| 0.97135 | 0.91527 | 0.99269 | 0.96637 | 0.93521 | Io__log__pm |
| 0.96711 | 0.91721 | 0.97403 | 0.95733 | 0.91841 | Io__cn1__log__pm |
| 0.97242 | 0.93243 | 0.97515 | 0.96268 | 0.92805 | Io__cn2__log__pm |
| 0.96801 | 0.90528 | 0.99312 | 0.96176 | 0.92662 | Io__cn1__cn2__log__pm |

#### **S2.3 JSRT → SH**

#### Table S26. Cross-dataset segmentation performance when training on JSRT and testing on SH using U-Net.

| Accuracy | precision | recall | dice | IoU | Experiment |
| --- | --- | --- | --- | --- | --- |
| 0.81650 | 0.64471 | 0.49384 | 0.52739 | 0.38308 | Io |
| 0.86156 | 0.73916 | 0.63796 | 0.66936 | 0.52178 | Io__cn1 |
| 0.92038 | 0.77869 | 0.92524 | 0.84981 | 0.74762 | Io__cn2 |
| 0.91994 | 0.81708 | 0.85093 | 0.83005 | 0.72177 | Io__cn1__cn2 |
| 0.89700 | 0.79857 | 0.75310 | 0.76450 | 0.63174 | Io__log |
| 0.90997 | 0.79211 | 0.83811 | 0.81265 | 0.69465 | Io__cn1__log |
| 0.92435 | 0.80151 | 0.90267 | 0.84922 | 0.74723 | Io__cn2__log |
| 0.92309 | 0.83358 | 0.84184 | 0.83258 | 0.72538 | Io__cn1__cn2__log |
| 0.95348 | 0.93377 | 0.86396 | 0.89051 | 0.80832 | Io__pm |
| 0.95493 | 0.87892 | 0.93806 | 0.91630 | 0.84853 | Io__cn1__pm |
| 0.95826 | 0.90698 | 0.91701 | 0.91313 | 0.84311 | Io__cn2__pm |
| 0.95496 | 0.89586 | 0.91536 | 0.91020 | 0.83824 | Io__cn1__cn2__pm |
| 0.95185 | 0.92472 | 0.86631 | 0.89145 | 0.80874 | Io__log__pm |
| 0.94893 | 0.92111 | 0.85676 | 0.88488 | 0.79938 | Io__cn1__log__pm |
| 0.95384 | 0.92936 | 0.87037 | 0.89317 | 0.81060 | Io__cn2__log__pm |
| 0.95772 | 0.87233 | 0.96138 | 0.92500 | 0.86307 | Io__cn1__cn2__log__pm |

#### Table S27. Cross-dataset segmentation performance when training on JSRT and testing on SH using ResUNet.

| accuracy | precision | recall | dice | IoU | Experiment |
| --- | --- | --- | --- | --- | --- |
| 0.96998 | 0.93141 | 0.94202 | 0.94023 | 0.88978 | Io |
| 0.96929 | 0.92654 | 0.94464 | 0.93929 | 0.88831 | Io__cn1 |
| 0.96905 | 0.92262 | 0.94828 | 0.94032 | 0.88969 | Io__cn2 |
| 0.96947 | 0.93020 | 0.94113 | 0.93929 | 0.88829 | Io__cn1__cn2 |
| 0.97025 | 0.92518 | 0.95072 | 0.94264 | 0.89365 | Io__log |
| 0.97048 | 0.92723 | 0.94931 | 0.94072 | 0.89074 | Io__cn1__log |
| 0.97050 | 0.93017 | 0.94588 | 0.94269 | 0.89358 | Io__cn2__log |
| 0.97114 | 0.92831 | 0.95105 | 0.94394 | 0.89566 | Io__cn1__cn2__log |
| 0.97193 | 0.92986 | 0.95279 | 0.94584 | 0.89924 | Io__pm |
| 0.97405 | 0.93822 | 0.95267 | 0.94875 | 0.90427 | Io__cn1__pm |
| 0.97354 | 0.93409 | 0.95518 | 0.94807 | 0.90276 | Io__cn2__pm |
| 0.97282 | 0.93114 | 0.95536 | 0.94730 | 0.90190 | Io__cn1__cn2__pm |
| 0.97085 | 0.92992 | 0.94778 | 0.94339 | 0.89499 | Io__log__pm |
| 0.97304 | 0.92888 | 0.95908 | 0.94983 | 0.90621 | Io__cn1__log__pm |
| 0.97372 | 0.93199 | 0.95849 | 0.95053 | 0.90754 | Io__cn2__log__pm |
| 0.97299 | 0.93257 | 0.95444 | 0.94818 | 0.90330 | Io__cn1__cn2__log__pm |

#### Table S28. Cross-dataset segmentation performance when training on JSRT and testing on SH using U-Net++.

| Accuracy | precision | recall | dice | IoU | Experiment |
| --- | --- | --- | --- | --- | --- |
| 0.90871 | 0.72975 | 0.97321 | 0.84092 | 0.73222 | Io |
| 0.97269 | 0.93715 | 0.94771 | 0.94542 | 0.89890 | Io__cn1 |
| 0.97290 | 0.93684 | 0.94904 | 0.94774 | 0.90292 | Io__cn2 |
| 0.97225 | 0.92981 | 0.95436 | 0.94492 | 0.89790 | Io__cn1__cn2 |
| 0.97213 | 0.93317 | 0.94982 | 0.94596 | 0.89962 | Io__log |
| 0.96858 | 0.93325 | 0.93350 | 0.93749 | 0.88503 | Io__cn1__log |
| 0.96554 | 0.93542 | 0.91715 | 0.92969 | 0.87155 | Io__cn2__log |
| 0.97085 | 0.92299 | 0.95614 | 0.94428 | 0.89655 | Io__cn1__cn2__log |
| 0.97238 | 0.93058 | 0.95404 | 0.94576 | 0.89880 | Io__pm |
| 0.97374 | 0.93179 | 0.95878 | 0.95016 | 0.90678 | Io__cn1__pm |
| 0.97086 | 0.91770 | 0.96276 | 0.94511 | 0.89838 | Io__cn2__pm |
| 0.97233 | 0.93552 | 0.94797 | 0.94485 | 0.89748 | Io__cn1__cn2__pm |
| 0.97344 | 0.92991 | 0.95970 | 0.94968 | 0.90644 | Io__log__pm |
| 0.97028 | 0.92769 | 0.94783 | 0.94438 | 0.89653 | Io__cn1__log__pm |
| 0.97111 | 0.91662 | 0.96527 | 0.94794 | 0.90296 | Io__cn2__log__pm |
| 0.97350 | 0.93790 | 0.95053 | 0.94716 | 0.90172 | Io__cn1__cn2__log__pm |

#### Table S29. Cross-dataset segmentation performance when training on JSRT and testing on SH using Attention Dense U-Net.

| Accuracy | precision | recall | dice | IoU | Experiment |
| --- | --- | --- | --- | --- | --- |
| 0.97281 | 0.92840 | 0.95860 | 0.94852 | 0.90394 | Io |
| 0.97021 | 0.92618 | 0.94932 | 0.94155 | 0.89208 | Io__cn1 |
| 0.97157 | 0.93271 | 0.94780 | 0.94432 | 0.89637 | Io__cn2 |
| 0.97407 | 0.93230 | 0.95972 | 0.95064 | 0.90751 | Io__cn1__cn2 |
| 0.97007 | 0.92343 | 0.95198 | 0.94172 | 0.89202 | Io__log |
| 0.97086 | 0.92155 | 0.95796 | 0.94535 | 0.89875 | Io__cn1__log |
| 0.97033 | 0.93036 | 0.94488 | 0.94189 | 0.89250 | Io__cn2__log |
| 0.97111 | 0.92106 | 0.95974 | 0.94489 | 0.89796 | Io__cn1__cn2__log |
| 0.97259 | 0.92904 | 0.95683 | 0.94773 | 0.90273 | Io__pm |
| 0.97203 | 0.92774 | 0.95582 | 0.94550 | 0.89881 | Io__cn1__pm |
| 0.97261 | 0.92853 | 0.95753 | 0.94856 | 0.90381 | Io__cn2__pm |
| 0.96932 | 0.91115 | 0.96387 | 0.94424 | 0.89679 | Io__cn1__cn2__pm |
| 0.97110 | 0.92140 | 0.95925 | 0.94655 | 0.90058 | Io__log__pm |
| 0.97131 | 0.92165 | 0.95990 | 0.94581 | 0.89930 | Io__cn1__log__pm |
| 0.97279 | 0.92772 | 0.95935 | 0.94894 | 0.90449 | Io__cn2__log__pm |
| 0.97188 | 0.92588 | 0.95737 | 0.94758 | 0.90224 | Io__cn1__cn2__log__pm |

#### Table S30. Cross-dataset segmentation performance when training on JSRT and testing on SH using Deep Attention U-Net.

| Accuracy | precision | recall | dice | IoU | Experiment |
| --- | --- | --- | --- | --- | --- |
| 0.96705 | 0.92667 | 0.93420 | 0.93229 | 0.87851 | Io |
| 0.97336 | 0.93319 | 0.95540 | 0.94670 | 0.90076 | Io__cn1 |
| 0.96645 | 0.92991 | 0.92763 | 0.92707 | 0.86780 | Io__cn2 |
| 0.96781 | 0.92467 | 0.94006 | 0.93635 | 0.88399 | Io__cn1__cn2 |
| 0.97261 | 0.92894 | 0.95702 | 0.94727 | 0.90203 | Io__log |
| 0.97206 | 0.92358 | 0.96100 | 0.94870 | 0.90421 | Io__cn1__log |
| 0.97001 | 0.92675 | 0.94769 | 0.93972 | 0.88932 | Io__cn2__log |
| 0.97204 | 0.92657 | 0.95729 | 0.94584 | 0.89938 | Io__cn1__cn2__log |
| 0.96840 | 0.90425 | 0.96853 | 0.94360 | 0.89542 | Io__pm |
| 0.97317 | 0.93292 | 0.95487 | 0.94794 | 0.90262 | Io__cn1__pm |
| 0.96561 | 0.92888 | 0.92497 | 0.93155 | 0.87576 | Io__cn2__pm |
| 0.97441 | 0.93844 | 0.95403 | 0.94992 | 0.90636 | Io__cn1__cn2__pm |
| 0.97068 | 0.93250 | 0.94395 | 0.94228 | 0.89317 | Io__log__pm |
| 0.97221 | 0.92947 | 0.95458 | 0.94780 | 0.90270 | Io__cn1__log__pm |
| 0.97091 | 0.92027 | 0.95975 | 0.94546 | 0.89921 | Io__cn2__log__pm |
| 0.97214 | 0.92454 | 0.96021 | 0.94706 | 0.90180 | Io__cn1__cn2__log__pm |

#### **S2.4 SH → JSRT**

#### Table S31. Cross-dataset segmentation performance when training on SH and testing on JSRT using U-Net.

| Accuracy | precision | recall | dice | iou | Experiment |
| --- | --- | --- | --- | --- | --- |
| 0.82175 | 0.83193 | 0.47960 | 0.59257 | 0.42910 | Io |
| 0.84541 | 0.74513 | 0.70619 | 0.73495 | 0.58726 | Io__cn1 |
| 0.90612 | 0.83813 | 0.83642 | 0.84608 | 0.73771 | Io__cn2 |
| 0.91166 | 0.85131 | 0.84095 | 0.85406 | 0.74933 | Io__cn1__cn2 |
| 0.91158 | 0.83807 | 0.85995 | 0.85676 | 0.75148 | Io__log |
| 0.91493 | 0.82999 | 0.88712 | 0.86897 | 0.76991 | Io__cn1__log |
| 0.91481 | 0.81076 | 0.91963 | 0.87617 | 0.78194 | Io__cn2__log |
| 0.92594 | 0.85768 | 0.89145 | 0.88339 | 0.79375 | Io__cn1__cn2__log |
| 0.95715 | 0.89586 | 0.96363 | 0.92875 | 0.86794 | Io__pm |
| 0.95934 | 0.90379 | 0.96154 | 0.93073 | 0.87139 | Io__cn1__pm |
| 0.95620 | 0.88995 | 0.96801 | 0.92964 | 0.86940 | Io__cn2__pm |
| 0.95529 | 0.88129 | 0.97673 | 0.93205 | 0.87373 | Io__cn1__cn2__pm |
| 0.95450 | 0.88189 | 0.97271 | 0.92828 | 0.86699 | Io__log__pm |
| 0.96090 | 0.90673 | 0.96374 | 0.93421 | 0.87742 | Io__cn1__log__pm |
| 0.95879 | 0.89585 | 0.97008 | 0.93477 | 0.87837 | Io__cn2__log__pm |
| 0.95349 | 0.87423 | 0.97988 | 0.93122 | 0.87223 | Io__cn1__cn2__log__pm |

#### Table S32. Cross-dataset segmentation performance when training on SH and testing on JSRT using ResUNet.

| Accuracy | Precision | recall | dice | iou | Experiment |
| --- | --- | --- | --- | --- | --- |
| 0.96770 | 0.94366 | 0.94454 | 0.94450 | 0.89781 | Io |
| 0.96319 | 0.94877 | 0.92231 | 0.93023 | 0.87423 | Io__cn1 |
| 0.94676 | 0.94275 | 0.86834 | 0.89330 | 0.82156 | Io__cn2 |
| 0.97280 | 0.95022 | 0.95587 | 0.95210 | 0.91005 | Io__cn1__cn2 |
| 0.96117 | 0.94468 | 0.91937 | 0.92903 | 0.87290 | Io__log |
| 0.95826 | 0.95144 | 0.90144 | 0.91739 | 0.85648 | Io__cn1__log |
| 0.95923 | 0.95204 | 0.90438 | 0.91853 | 0.85704 | Io__cn2__log |
| 0.94581 | 0.94261 | 0.86498 | 0.89224 | 0.81716 | Io__cn1__cn2__log |
| 0.97278 | 0.94165 | 0.96555 | 0.95466 | 0.91482 | Io__pm |
| 0.96853 | 0.94549 | 0.94554 | 0.94255 | 0.89521 | Io__cn1__pm |
| 0.97135 | 0.94958 | 0.95130 | 0.94597 | 0.90056 | Io__cn2__pm |
| 0.97585 | 0.94283 | 0.97553 | 0.96093 | 0.92548 | Io__cn1__cn2__pm |
| 0.92316 | 0.94694 | 0.77745 | 0.82224 | 0.72914 | Io__log__pm |
| 0.97763 | 0.95229 | 0.97117 | 0.96052 | 0.92464 | Io__cn1__log__pm |
| 0.97425 | 0.94849 | 0.96314 | 0.95470 | 0.91501 | Io__cn2__log__pm |
| 0.96496 | 0.94552 | 0.93237 | 0.93572 | 0.88364 | Io__cn1__cn2__log__pm |

#### Table S33. Cross-dataset segmentation performance when training on SH and testing on JSRT using U-Net++

| Accuracy | Precision | recall | dice | iou | Experiment |
| --- | --- | --- | --- | --- | --- |
| 0.94157 | 0.94544 | 0.84650 | 0.87616 | 0.79812 | Io |
| 0.95200 | 0.92849 | 0.90334 | 0.91223 | 0.84810 | Io__cn1 |
| 0.97172 | 0.94022 | 0.96330 | 0.95241 | 0.91026 | Io__cn2 |
| 0.97131 | 0.93206 | 0.97146 | 0.95469 | 0.91449 | Io__cn1__cn2 |
| 0.97091 | 0.92408 | 0.97975 | 0.95963 | 0.92305 | Io__log |
| 0.95361 | 0.93418 | 0.90298 | 0.91656 | 0.85322 | Io__cn1__log |
| 0.96973 | 0.92580 | 0.97318 | 0.95458 | 0.91392 | Io__cn2__log |
| 0.97372 | 0.92907 | 0.98411 | 0.96121 | 0.92578 | Io__cn1__cn2__log |
| 0.97263 | 0.96174 | 0.94270 | 0.94856 | 0.90348 | Io__pm |
| 0.97323 | 0.93856 | 0.97085 | 0.95524 | 0.91580 | Io__cn1__pm |
| 0.97239 | 0.93041 | 0.97750 | 0.96005 | 0.92402 | Io__cn2__pm |
| 0.96793 | 0.94106 | 0.94835 | 0.94547 | 0.89876 | Io__cn1__cn2__pm |
| 0.95160 | 0.94119 | 0.88784 | 0.90443 | 0.83644 | Io__log__pm |
| 0.95399 | 0.95255 | 0.88473 | 0.90620 | 0.83833 | Io__cn1__log__pm |
| 0.96478 | 0.94269 | 0.93488 | 0.93649 | 0.88442 | Io__cn2__log__pm |
| 0.96616 | 0.93380 | 0.95016 | 0.94322 | 0.89628 | Io__cn1__cn2__log__pm |

#### Table S34. Cross-dataset segmentation performance when training on SH and testing on JSRT using Attention Dense U-Net

| Accuracy | precision | recall | dice | iou | Experiment |
| --- | --- | --- | --- | --- | --- |
| 0.96850 | 0.92658 | 0.96757 | 0.95012 | 0.90605 | Io |
| 0.96820 | 0.92744 | 0.96541 | 0.94727 | 0.90182 | Io__cn1 |
| 0.95914 | 0.93665 | 0.92078 | 0.92538 | 0.86627 | Io__cn2 |
| 0.96737 | 0.94000 | 0.94746 | 0.94211 | 0.89206 | Io__cn1__cn2 |
| 0.96649 | 0.93675 | 0.94798 | 0.94287 | 0.89510 | Io__log |
| 0.96980 | 0.94003 | 0.95643 | 0.94755 | 0.90260 | Io__cn1__log |
| 0.96147 | 0.92582 | 0.94206 | 0.93522 | 0.88194 | Io__cn2__log |
| 0.97140 | 0.93956 | 0.96290 | 0.94996 | 0.90664 | Io__cn1__cn2__log |
| 0.96312 | 0.93110 | 0.94197 | 0.93575 | 0.88335 | Io__pm |
| 0.97346 | 0.94375 | 0.96564 | 0.95337 | 0.91206 | Io__cn1__pm |
| 0.96714 | 0.93660 | 0.95053 | 0.94261 | 0.89483 | Io__cn2__pm |
| 0.96644 | 0.94052 | 0.94346 | 0.94032 | 0.88989 | Io__cn1__cn2__pm |
| 0.96235 | 0.93252 | 0.93746 | 0.93348 | 0.87863 | Io__log__pm |
| 0.95500 | 0.94504 | 0.89627 | 0.91248 | 0.84536 | Io__cn1__log__pm |
| 0.95977 | 0.94272 | 0.91636 | 0.92390 | 0.86358 | Io__cn2__log__pm |
| 0.95774 | 0.92915 | 0.92411 | 0.92517 | 0.86521 | Io__cn1__cn2__log__pm |

#### Table S35. Cross-dataset segmentation performance when training on SH and testing on JSRT using Deep Attention U-Net

| Accuracy | precision | recall | Dice | IoU | Experiment |
| --- | --- | --- | --- | --- | --- |
| 0.97296 | 0.93430 | 0.97490 | 0.95763 | 0.91957 | Io |
| 0.96916 | 0.93595 | 0.95881 | 0.94940 | 0.90696 | Io__cn1 |
| 0.95894 | 0.93293 | 0.92424 | 0.92643 | 0.86771 | Io__cn2 |
| 0.96143 | 0.92049 | 0.94834 | 0.93654 | 0.88298 | Io__cn1__cn2 |
| 0.91403 | 0.92783 | 0.76149 | 0.81740 | 0.72043 | Io__log |
| 0.94441 | 0.91066 | 0.89531 | 0.90435 | 0.83458 | Io__cn1__log |
| 0.96094 | 0.92124 | 0.94557 | 0.93729 | 0.88648 | Io__cn2__log |
| 0.94145 | 0.90490 | 0.89085 | 0.90365 | 0.83181 | Io__cn1__cn2__log |
| 0.96730 | 0.93275 | 0.95567 | 0.94358 | 0.89633 | Io__pm |
| 0.97156 | 0.93489 | 0.96900 | 0.95510 | 0.91546 | Io__cn1__pm |
| 0.96934 | 0.92212 | 0.97626 | 0.95413 | 0.91385 | Io__cn2__pm |
| 0.97009 | 0.92450 | 0.97612 | 0.95389 | 0.91294 | Io__cn1__cn2__pm |
| 0.97230 | 0.93521 | 0.97137 | 0.95844 | 0.92128 | Io__log__pm |
| 0.96878 | 0.92274 | 0.97339 | 0.95409 | 0.91321 | Io__cn1__log__pm |
| 0.97204 | 0.93477 | 0.97092 | 0.95980 | 0.92249 | Io__cn2__log__pm |
| 0.97164 | 0.92883 | 0.97664 | 0.95811 | 0.92065 | Io__cn1__cn2__log__pm |

#### **S2.5 MC → SH**

#### Table S36. Cross-dataset segmentation performance when training on MC and testing on SH using

#### U-Net.

| Accuracy | precision | recall | dice | IoU | Experiment |
| --- | --- | --- | --- | --- | --- |
| 0.75348 | 0.44649 | 0.19020 | 0.22111 | 0.14910 | Io |
| 0.79011 | 0.59285 | 0.35050 | 0.40848 | 0.28458 | Io__cn1 |
| 0.91912 | 0.80403 | 0.86866 | 0.83538 | 0.72425 | Io__cn2 |
| 0.90748 | 0.88100 | 0.70246 | 0.76721 | 0.63301 | Io__cn1__cn2 |
| 0.84592 | 0.72761 | 0.55378 | 0.61025 | 0.46425 | Io__log |
| 0.84450 | 0.72264 | 0.55253 | 0.61286 | 0.46546 | Io__cn1__log |
| 0.91195 | 0.87798 | 0.72768 | 0.78405 | 0.65488 | Io__cn2__log |
| 0.93642 | 0.88030 | 0.84528 | 0.86244 | 0.76134 | Io__cn1__cn2__log |
| 0.95632 | 0.87885 | 0.94500 | 0.91515 | 0.84650 | Io__pm |
| 0.92196 | 0.91851 | 0.73411 | 0.80135 | 0.68614 | Io__cn1__pm |
| 0.94503 | 0.87345 | 0.89681 | 0.89224 | 0.80826 | Io__cn2__pm |
| 0.94854 | 0.89304 | 0.88809 | 0.89326 | 0.81022 | Io__cn1__cn2__pm |
| 0.93836 | 0.89065 | 0.84194 | 0.86666 | 0.76902 | Io__log__pm |
| 0.94450 | 0.90263 | 0.85705 | 0.87694 | 0.78532 | Io__cn1__log__pm |
| 0.95173 | 0.87773 | 0.92400 | 0.90706 | 0.83205 | Io__cn2__log__pm |
| 0.94785 | 0.87423 | 0.90968 | 0.89851 | 0.81831 | Io__cn1__cn2__log__pm |

#### Table S37. Cross-dataset segmentation performance when training on MC and testing on SH using ResUNet.

| Accuracy | precision | recall | Dice | IoU | Experiment |
| --- | --- | --- | --- | --- | --- |
| 0.95698 | 0.88561 | 0.93877 | 0.92208 | 0.86001 | Io |
| 0.94622 | 0.89622 | 0.87300 | 0.88655 | 0.80994 | Io__cn1 |
| 0.94114 | 0.91729 | 0.82472 | 0.86528 | 0.78171 | Io__cn2 |
| 0.95873 | 0.88511 | 0.94804 | 0.92669 | 0.86730 | Io__cn1__cn2 |
| 0.96192 | 0.89893 | 0.94469 | 0.93249 | 0.87718 | Io__log |
| 0.95663 | 0.88138 | 0.94295 | 0.92197 | 0.85946 | Io__cn1__log |
| 0.96092 | 0.89410 | 0.94633 | 0.93142 | 0.87559 | Io__cn2__log |
| 0.95890 | 0.87277 | 0.96660 | 0.93177 | 0.87471 | Io__cn1__cn2__log |
| 0.95748 | 0.88734 | 0.93885 | 0.92510 | 0.86558 | Io__pm |
| 0.95508 | 0.88745 | 0.92703 | 0.91875 | 0.85548 | Io__cn1__pm |
| 0.96293 | 0.89517 | 0.95456 | 0.93590 | 0.88267 | Io__cn2__pm |
| 0.95858 | 0.89561 | 0.93306 | 0.92605 | 0.86667 | Io__cn1__cn2__pm |
| 0.95843 | 0.89565 | 0.93229 | 0.92451 | 0.86571 | Io__log__pm |
| 0.95960 | 0.87691 | 0.96395 | 0.93171 | 0.87459 | Io__cn1__log__pm |
| 0.96138 | 0.88273 | 0.96432 | 0.93585 | 0.88145 | Io__cn2__log__pm |
| 0.96343 | 0.89017 | 0.96381 | 0.93685 | 0.88358 | Io__cn1__cn2__log__pm |

#### Table S38. Cross-dataset segmentation performance when training on MC and testing on SH using U-Net++.

| Accuracy | precision | recall | dice | IoU | Experiment |
| --- | --- | --- | --- | --- | --- |
| 0.96140 | 0.88843 | 0.95640 | 0.93387 | 0.87874 | Io |
| 0.96576 | 0.90528 | 0.95466 | 0.93945 | 0.88832 | Io__cn1 |
| 0.96537 | 0.90683 | 0.95082 | 0.93780 | 0.88650 | Io__cn2 |
| 0.96258 | 0.88452 | 0.96763 | 0.93810 | 0.88522 | Io__cn1__cn2 |
| 0.96570 | 0.90028 | 0.96097 | 0.94222 | 0.89293 | Io__log |
| 0.95694 | 0.87666 | 0.95121 | 0.92438 | 0.86293 | Io__cn1__log |
| 0.96216 | 0.87948 | 0.97281 | 0.93777 | 0.88452 | Io__cn2__log |
| 0.94873 | 0.88564 | 0.89859 | 0.90439 | 0.82859 | Io__cn1__cn2__log |
| 0.96279 | 0.88460 | 0.96853 | 0.93748 | 0.88525 | Io__pm |
| 0.96018 | 0.87303 | 0.97255 | 0.93403 | 0.87868 | Io__cn1__pm |
| 0.96316 | 0.88867 | 0.96460 | 0.93909 | 0.88740 | Io__cn2__pm |
| 0.96520 | 0.89264 | 0.96895 | 0.94243 | 0.89293 | Io__cn1__cn2__pm |
| 0.96008 | 0.88448 | 0.95549 | 0.93121 | 0.87488 | Io__log__pm |
| 0.96531 | 0.90506 | 0.95283 | 0.94123 | 0.89141 | Io__cn1__log__pm |
| 0.96312 | 0.88929 | 0.96352 | 0.93864 | 0.88635 | Io__cn2__log__pm |
| 0.96183 | 0.88390 | 0.96485 | 0.93734 | 0.88446 | Io__cn1__cn2__log__pm |

#### Table S39. Cross-dataset segmentation performance when training on MC and testing on SH using

#### Attention Dense U-Net.

| Accuracy | precision | recall | dice | IoU | Experiment |
| --- | --- | --- | --- | --- | --- |
| 0.94591 | 0.89376 | 0.87453 | 0.88972 | 0.81321 | Io |
| 0.94993 | 0.86069 | 0.93973 | 0.90607 | 0.83552 | Io__cn1 |
| 0.95060 | 0.85539 | 0.95132 | 0.91186 | 0.84430 | Io__cn2 |
| 0.92388 | 0.91382 | 0.74767 | 0.81116 | 0.70656 | Io__cn1__cn2 |
| 0.94483 | 0.86169 | 0.91243 | 0.89540 | 0.81992 | Io__log |
| 0.96147 | 0.89080 | 0.95346 | 0.93462 | 0.88019 | Io__cn1__log |
| 0.95038 | 0.89820 | 0.89044 | 0.89979 | 0.82915 | Io__cn2__log |
| 0.94887 | 0.83885 | 0.96934 | 0.91190 | 0.84224 | Io__cn1__cn2__log |
| 0.95642 | 0.87706 | 0.94808 | 0.92503 | 0.86549 | Io__pm |
| 0.96523 | 0.89758 | 0.96234 | 0.94199 | 0.89312 | Io__cn1__pm |
| 0.95835 | 0.88684 | 0.94378 | 0.92728 | 0.86886 | Io__cn2__pm |
| 0.95579 | 0.88488 | 0.93401 | 0.92197 | 0.86072 | Io__cn1__cn2__pm |
| 0.96122 | 0.87920 | 0.96859 | 0.93645 | 0.88258 | Io__log__pm |
| 0.96528 | 0.89453 | 0.96674 | 0.94219 | 0.89270 | Io__cn1__log__pm |
| 0.96214 | 0.89491 | 0.95110 | 0.93574 | 0.88090 | Io__cn2__log__pm |
| 0.94660 | 0.89769 | 0.87299 | 0.89399 | 0.81759 | Io__cn1__cn2__log__pm |

#### Table S40. Cross-dataset segmentation performance when training on MC and testing on SH using Deep Attention U-Net.

| Accuracy | precision | recall | dice | IoU | Experiment |
| --- | --- | --- | --- | --- | --- |
| 0.96208 | 0.88264 | 0.96783 | 0.93694 | 0.88334 | Io |
| 0.96350 | 0.89520 | 0.95724 | 0.93667 | 0.88348 | Io__cn1 |
| 0.95972 | 0.88647 | 0.95095 | 0.92805 | 0.86865 | Io__cn2 |
| 0.96513 | 0.89168 | 0.96994 | 0.94136 | 0.89082 | Io__cn1__cn2 |
| 0.96318 | 0.89816 | 0.95177 | 0.93411 | 0.87996 | Io__log |
| 0.96274 | 0.88197 | 0.97205 | 0.93794 | 0.88475 | Io__cn1__log |
| 0.95736 | 0.87374 | 0.95754 | 0.92749 | 0.86800 | Io__cn2__log |
| 0.95342 | 0.86724 | 0.94749 | 0.91794 | 0.85324 | Io__cn1__cn2__log |
| 0.96208 | 0.90580 | 0.93655 | 0.92900 | 0.87083 | Io__pm |
| 0.95982 | 0.89360 | 0.94169 | 0.92856 | 0.87118 | Io__cn1__pm |
| 0.95985 | 0.87120 | 0.97368 | 0.93270 | 0.87632 | Io__cn2__pm |
| 0.96338 | 0.89379 | 0.95857 | 0.93754 | 0.88489 | Io__cn1__cn2__pm |
| 0.95873 | 0.86860 | 0.97199 | 0.93082 | 0.87264 | Io__log__pm |
| 0.96042 | 0.88589 | 0.95517 | 0.93156 | 0.87521 | Io__cn1__log__pm |
| 0.96616 | 0.89901 | 0.96483 | 0.94226 | 0.89280 | Io__cn2__log__pm |
| 0.96443 | 0.88786 | 0.97189 | 0.94090 | 0.89018 | Io__cn1__cn2__log__pm |

#### **S2.6 MC → SH**

#### Table S41. Cross-dataset segmentation performance when training on SH and testing on MC using U-Net.

| Accuracy | Precision | recall | dice | IoU | Experiment |
| --- | --- | --- | --- | --- | --- |
| 0.79372 | 0.65297 | 0.38843 | 0.47787 | 0.32083 | Io |
| 0.83889 | 0.69185 | 0.65112 | 0.67991 | 0.51915 | Io__cn1 |
| 0.87732 | 0.80083 | 0.68351 | 0.72892 | 0.58790 | Io__cn2 |
| 0.87952 | 0.81250 | 0.67893 | 0.74213 | 0.59819 | Io__cn1__cn2 |
| 0.88566 | 0.78799 | 0.74780 | 0.76102 | 0.62408 | Io__log |
| 0.90510 | 0.81234 | 0.81103 | 0.81521 | 0.69395 | Io__cn1__log |
| 0.90070 | 0.83124 | 0.76066 | 0.78875 | 0.66201 | Io__cn2__log |
| 0.91112 | 0.85052 | 0.78561 | 0.81293 | 0.69365 | Io__cn1__cn2__log |
| 0.96045 | 0.92522 | 0.91730 | 0.88711 | 0.82050 | Io__pm |
| 0.95868 | 0.90647 | 0.93236 | 0.89356 | 0.82720 | Io__cn1__pm |
| 0.95831 | 0.91587 | 0.91908 | 0.88585 | 0.81749 | Io__cn2__pm |
| 0.96034 | 0.94161 | 0.89844 | 0.88040 | 0.80988 | Io__cn1__cn2__pm |
| 0.95907 | 0.91089 | 0.92853 | 0.89027 | 0.82454 | Io__log__pm |
| 0.95889 | 0.90635 | 0.93344 | 0.89432 | 0.82828 | Io__cn1__log__pm |
| 0.96050 | 0.91663 | 0.92776 | 0.89226 | 0.82753 | Io__cn2__log__pm |
| 0.95754 | 0.89880 | 0.93712 | 0.89280 | 0.82768 | Io__cn1__cn2__log__pm |

#### Table S42. Cross-dataset segmentation performance when training on SH and testing on MC using ResUNet.

| Accuracy | precision | recall | dice | IoU | Experiment |
| --- | --- | --- | --- | --- | --- |
| 0.96179 | 0.95839 | 0.88700 | 0.90575 | 0.83841 | Io |
| 0.96738 | 0.96714 | 0.90125 | 0.91919 | 0.85734 | Io__cn1 |
| 0.95880 | 0.95593 | 0.87705 | 0.89651 | 0.82254 | Io__cn2 |
| 0.96348 | 0.96462 | 0.88772 | 0.90495 | 0.83932 | Io__cn1__cn2 |
| 0.93067 | 0.95839 | 0.75798 | 0.82396 | 0.72505 | Io__log |
| 0.93133 | 0.96722 | 0.75323 | 0.81914 | 0.72429 | Io__cn1__log |
| 0.96412 | 0.96098 | 0.89403 | 0.91581 | 0.84950 | Io__cn2__log |
| 0.97133 | 0.96302 | 0.92172 | 0.93102 | 0.87447 | Io__cn1__cn2__log |
| 0.96449 | 0.96921 | 0.88739 | 0.90158 | 0.83314 | Io__pm |
| 0.95792 | 0.96503 | 0.86445 | 0.88927 | 0.81343 | Io__cn1__pm |
| 0.96770 | 0.96775 | 0.90198 | 0.91314 | 0.84766 | Io__cn2__pm |
| 0.95102 | 0.96831 | 0.83305 | 0.87069 | 0.78288 | Io__cn1__cn2__pm |
| 0.96067 | 0.97467 | 0.86656 | 0.86912 | 0.79891 | Io__log__pm |
| 0.96901 | 0.96668 | 0.90842 | 0.91045 | 0.84690 | Io__cn1__log__pm |
| 0.97928 | 0.96943 | 0.94772 | 0.94202 | 0.89469 | Io__cn2__log__pm |
| 0.96980 | 0.96564 | 0.91272 | 0.91645 | 0.85365 | Io__cn1__cn2__log__pm |

#### Table S43. Cross-dataset segmentation performance when training on SH and testing on MC using U-Net++.

| accuracy | precision | recall | dice | IoU | Experiment |
| --- | --- | --- | --- | --- | --- |
| 0.89604 | 0.95778 | 0.61485 | 0.73037 | 0.60604 | Io |
| 0.94471 | 0.95393 | 0.82035 | 0.85294 | 0.76887 | Io__cn1 |
| 0.96991 | 0.94198 | 0.93849 | 0.93270 | 0.87839 | Io__cn2 |
| 0.96919 | 0.96031 | 0.91565 | 0.92414 | 0.86526 | Io__cn1__cn2 |
| 0.91124 | 0.96236 | 0.67439 | 0.76310 | 0.63797 | Io__log |
| 0.97323 | 0.95530 | 0.93773 | 0.93800 | 0.88616 | Io__cn1__log |
| 0.96310 | 0.95015 | 0.90093 | 0.91483 | 0.84722 | Io__cn2__log |
| 0.92284 | 0.94937 | 0.73310 | 0.80023 | 0.69926 | Io__cn1__cn2__log |
| 0.96216 | 0.96009 | 0.88680 | 0.91023 | 0.84268 | Io__pm |
| 0.95176 | 0.96675 | 0.83752 | 0.86276 | 0.78296 | Io__cn1__pm |
| 0.96487 | 0.95322 | 0.90510 | 0.92178 | 0.85962 | Io__cn2__pm |
| 0.96889 | 0.95963 | 0.91514 | 0.92051 | 0.86005 | Io__cn1__cn2__pm |
| 0.97688 | 0.96789 | 0.93947 | 0.93117 | 0.88142 | Io__log__pm |
| 0.97176 | 0.95124 | 0.93600 | 0.93355 | 0.87794 | Io__cn1__log__pm |
| 0.96717 | 0.95843 | 0.90926 | 0.91451 | 0.85239 | Io__cn2__log__pm |
| 0.94364 | 0.96755 | 0.80344 | 0.86009 | 0.76470 | Io__cn1__cn2__log__pm |

#### Table S44. Cross-dataset segmentation performance when training on SH and testing on MC using Attention Dense U-Net.

| Accuracy | Precision | recall | dice | IoU | Experiment |
| --- | --- | --- | --- | --- | --- |
| 0.96050 | 0.95374 | 0.88633 | 0.90325 | 0.82941 | Io |
| 0.96288 | 0.94505 | 0.90544 | 0.92060 | 0.85699 | Io__cn1 |
| 0.95646 | 0.94198 | 0.88163 | 0.89526 | 0.82218 | Io__cn2 |
| 0.95619 | 0.95473 | 0.86741 | 0.89194 | 0.81395 | Io__cn1__cn2 |
| 0.97113 | 0.94752 | 0.93744 | 0.93450 | 0.87942 | Io__log |
| 0.93934 | 0.94587 | 0.80554 | 0.85293 | 0.75923 | Io__cn1__log |
| 0.95232 | 0.96088 | 0.84533 | 0.88030 | 0.79889 | Io__cn2__log |
| 0.96575 | 0.95073 | 0.91140 | 0.91293 | 0.85011 | Io__cn1__cn2__log |
| 0.95142 | 0.96121 | 0.84131 | 0.87448 | 0.79320 | Io__pm |
| 0.96878 | 0.95901 | 0.91533 | 0.91677 | 0.85329 | Io__cn1__pm |
| 0.96389 | 0.95001 | 0.90440 | 0.90325 | 0.83500 | Io__cn2__pm |
| 0.97726 | 0.96449 | 0.94460 | 0.94089 | 0.89115 | Io__cn1__cn2__pm |
| 0.96673 | 0.95772 | 0.90815 | 0.91837 | 0.85486 | Io__log__pm |
| 0.97724 | 0.95591 | 0.95373 | 0.94321 | 0.89537 | Io__cn1__log__pm |
| 0.95147 | 0.96920 | 0.83405 | 0.87173 | 0.78778 | Io__cn2__log__pm |
| 0.96061 | 0.95381 | 0.88674 | 0.90131 | 0.82650 | Io__cn1__cn2__log__pm |

#### Table S45. Cross-dataset segmentation performance when training on SH and testing on MC using Deep Attention U-Net.

| Accuracy | precision | recall | dice | IoU | Experiment |
| --- | --- | --- | --- | --- | --- |
| 0.97051 | 0.94161 | 0.94144 | 0.93563 | 0.88335 | Io |
| 0.94128 | 0.94355 | 0.81596 | 0.86539 | 0.77556 | Io__cn1 |
| 0.94946 | 0.95074 | 0.84327 | 0.88727 | 0.80747 | Io__cn2 |
| 0.95197 | 0.96049 | 0.84427 | 0.88737 | 0.81195 | Io__cn1__cn2 |
| 0.90168 | 0.96168 | 0.63541 | 0.72945 | 0.61453 | Io__log |
| 0.96149 | 0.94873 | 0.89569 | 0.91902 | 0.85490 | Io__cn1__log |
| 0.93542 | 0.94437 | 0.79047 | 0.85314 | 0.76284 | Io__cn2__log |
| 0.96928 | 0.94061 | 0.93736 | 0.93179 | 0.87432 | Io__cn1__cn2__log |
| 0.97192 | 0.94661 | 0.94179 | 0.93615 | 0.88261 | Io__pm |
| 0.96556 | 0.94428 | 0.91757 | 0.92481 | 0.86418 | Io__cn1__pm |
| 0.94673 | 0.94929 | 0.83325 | 0.87753 | 0.79262 | Io__cn2__pm |
| 0.93233 | 0.92563 | 0.79556 | 0.84325 | 0.74005 | Io__cn1__cn2__pm |
| 0.96915 | 0.96539 | 0.91028 | 0.91658 | 0.85437 | Io__log__pm |
| 0.97863 | 0.95693 | 0.95840 | 0.95151 | 0.90915 | Io__cn1__log__pm |
| 0.98283 | 0.96956 | 0.96212 | 0.95562 | 0.91674 | Io__cn2__log__pm |
| 0.92896 | 0.95894 | 0.75044 | 0.83275 | 0.72552 | Io__cn1__cn2__log__pm |
